# The effectiveness of computerised decision support on antibiotic use in hospitals: A systematic review

**DOI:** 10.1371/journal.pone.0183062

**Published:** 2017-08-24

**Authors:** Christopher E. Curtis, Fares Al Bahar, John F. Marriott

**Affiliations:** 1 School of Pharmacy, College of Medical & Dental Sciences, University of Birmingham, Birmingham, United Kingdom; 2 University Hospitals Birmingham NHS Foundation Trust, Edgbaston, Birmingham, United Kingdom; Cairo University, EGYPT

## Abstract

**Background:**

Inappropriate antimicrobial use has been shown to be an important determinant of the emergence of antimicrobial resistance (AMR). Health information technology (HIT) in the form of Computerised Decision Support (CDS) represents an option for improving antimicrobial prescribing and containing AMR.

**Objectives:**

To evaluate the evidence for CDS in improving quantitative and qualitative measures of antibiotic prescribing in inpatient hospital settings.

**Methods:**

A systematic literature search was conducted of articles published from inception to 20^th^ December 2014 using eight electronic databases: MEDLINE, EMBASE, PUBMED, Web of Science, CINAHL, Cochrane Library, HMIC and PsychINFo. An updated systematic literature search was conducted from January 1^st^ 2015 to October 1^st^ 2016 using PUBMED. The search strategy used combinations of the following terms: (electronic prescribing) OR (clinical decision support) AND (antibiotic or antibacterial or antimicrobial) AND (hospital or secondary care or inpatient). Studies were evaluated for quality using a 10-point rating scale.

**Results:**

Eighty-one studies were identified matching the inclusion criteria. Seven outcome measures were evaluated: adequacy of antibiotic coverage, mortality, volume of antibiotic usage, length of stay, antibiotic cost, compliance with guidelines, antimicrobial resistance, and CDS implementation and uptake. Meta-analysis of pooled outcomes showed CDS significantly improved the adequacy of antibiotic coverage (n = 13; odds ratio [OR], 2.11 [95% CI, 1.67 to 2.66, p ≤ 0.00001]). Also, CDS was associated with marginally lowered mortality (n = 20; OR, 0.85 [CI, 0.75 to 0.96, p = 0.01]). CDS was associated with lower antibiotic utilisation, increased compliance with antibiotic guidelines and reductions in antimicrobial resistance. Conflicting effects of CDS on length of stay, antibiotic costs and system uptake were also noted.

**Conclusions:**

CDS has the potential to improve the adequacy of antibiotic coverage and marginally decrease mortality in hospital-related settings.

## Introduction

Antimicrobials have saved millions of lives since their introduction[1] however; antimicrobial resistance (AMR) has increased over the past four decades.[2] Evidence shows that 30%-50% of antimicrobial prescribing is sub-optimal.[3] Inappropriate antimicrobial use has been shown to be an important determinant of the emergence and persistence of AMR.[2] This pattern of irrational use in hospitals and the relative reduction in development of new antibiotic entities pose a challenge for clinicians, as their options to treat infections, especially those caused by resistant pathogens, become limited.

The use of health information technology (HIT) is one strategy to optimise antibiotic use in health care settings. Over the last twenty years, there have been rapid advances and investment in HIT, manifesting as an increased uptake of the use of computers in healthcare. The NHS embraces the role of HIT in optimising the quality of care and patient safety. In the UK, £12.8 billion has been invested in the National Programme for Information Technology (NPfIT) by the National Health Service (NHS).[4] Computerised Decision Support (CDS) represents a potential solution for improving antimicrobial prescribing and containing antimicrobial resistance by supporting clinical decision making[5,6] thus optimising antibiotic use and improving patient outcomes. It potentially plays an important role in guiding prescribing practices such as antibiotic selection and dosing suggestions, alerting potential adverse drug reactions and drug allergies.

Two previous systematic reviews focused on the impact CDS on antibiotic use in primary care[7] and included non-computerised decision support.[8] Another more recent systematic review addressed a similar research question and examined the impact of HIT interventions on antimicrobial prescribing.[9] The scope, design and timing of these reviews may have excluded relevant CDS studies that match the inclusion criteria in this review. The aim of the present study was to evaluate the current state of evidence for CDS interventions on antibiotic use in the hospital inpatient setting. Meta-analysis was conducted using odds ratio to assess the impact of CDS on the adequacy of antibiotic coverage and mortality and to assess the impact of CDS, using relative differences, on length of stay, volume of antibiotic use, antimicrobial resistance and compliance with guidelines.

## Methods

### Data source and study selection

A systematic literature search was conducted utilising eight online databases including MEDLINE, EMBASE, PUBMED, Web of Science, CINAHL, Cochrane Library, HMIC, and PsycINFO. The search was conducted from inception to 20^th^ December, 2014. An updated literature search was conducted from January 1^st^ 2015 to October 1^st^ 2016 using PUBMED. The searches were conducted using a strategy based upon combinations of the following terms: (electronic prescribing) OR (clinical decision support) AND (antibiotic or antibacterial or antimicrobial) AND (hospital or secondary care or inpatient). The search strategy appears in S1 Appendix, PRISMA search strategy details. This was supported by use of a checklist S1 Checklist PRISMA 2009 checklist to ensure that PRISMA principles were followed during the process.

Titles and abstracts from retrieved references were examined by two reviewers (FA and CEC) to determine the potential inclusion eligibility. Full texts of potential studies were examined for eligibility against the review inclusion criteria. Bibliographies of retrieved articles and previous systematic reviews were examined to identify additional articles that could have been missed by this search strategy.

### Inclusion and exclusion criteria

Criteria for inclusion in the systematic review were: (i) conducted by health care providers in inpatient or ICU or emergency (ED) settings (ii) the intervention involved CDS aimed at improving antibiotic prescribing at the point of care and (iii) the intervention was compared to no intervention, non-CDS intervention (non-electronic decision support) or to an intervention with CDS of different features. For the purpose of the systematic review, CDS was defined as a computer-based system designed to help directly in clinical decision making in which characteristics of individual patients are utilised to generate recommendations presented to clinicians at the point of care in a passive or active format such as alerts, reminders and guidelines.[10–12]

Non-electronic CDS studies, non-hospital based studies, qualitative studies, case reports, case series studies, conference abstracts, commentaries, and letters, papers examining the performance of the system as opposed to its impact on antibiotic prescribing were excluded. In the case where a study had an unclear inclusion status, conflicts were resolved and consensus was reached by a third reviewer (JFM).

### Data extraction and quality assessment

A custom data extraction form was created to match the specific needs of the review. Data related to study design, participants, interventions, comparators, outcomes, and main findings were extracted by one reviewer (FA) and confirmed by another (CEC). Disagreements were resolved by consensus, with a third investigator (JFM). When studies did not report sufficient data to allow pooling for meta-analysis, results were summarised qualitatively using relative differences. Email requests for additional data were made to authors of papers containing insufficient information to be included in the meta-analyses.

The quality of included studies was assessed using a 10-point rating scale previously used to evaluate CDS studies (see Table 1).[9,13–15] The scale included five domains (2 points per domain): method of allocation of study groups, unit of allocation, presence of baseline differences between groups, objectivity of outcome measures, and completeness of follow-up for appropriate unit of analysis. Assessment of the methodological quality of the eligible studies was undertaken independently by two reviewers (FA, CEC). Reviewer disagreements were resolved by a third reviewer (JFM).

**Table 1:** Detail of the 10-point Quality Assessment Scale used in the present study.

**1. Method of allocation of study groups** 2 = Random, 1 = Quasi-random, ) = Selected concurrent controls
**2. Unit of allocation** 2 = Cluster (e.g. practice), 1 = Physician, 0 = Patients
**3. Presence of baseline differences between groups** 2 = No baseline differences present or appropriate statistical adjustments made 1 = Baseline differences present and no statistical adjustment made 0 = Baseline characteristics not reported
**4. Objectivity of outcome measures** **2** = Objective outcomes or subjective outcomes with blinded assessment 1 = Subjective outcomes with no blinding but clearly defined assessment criteria 0 = Subjective outcomes with no blinding and poorly defined assessment
**5. Completeness of follow up for appropriate unit of analysis** 2 = >90%, 1 = 80–90%, 0 = <80%

### Data analysis and statistical analysis

A defined set of outcomes essential in estimating the effect of CDS in optimising antibiotic use shaped the synthesis process. Meta-analysis was conducted when studies evaluated the same outcome and had sufficient data to allow pooling. All studies were eligible for consideration for inclusion in the meta-analysis as all assessed the impact of CDS on antibiotic prescribing in the hospital inpatient setting. The meta-analysis focused on two outcomes: adequacy of antibiotic coverage (13 studies) and mortality (20 studies). Odds ratios and 95% confidence intervals (CIs) were calculated for each trial by reconstructing tables based on the number of patients randomly allocated and the number of patients with the outcome of interest. Inter-study variance was assessed using the Tau^2^ test. Inter-study heterogeneity was assessed using the Chi^2^ test and the *I*^*2*^ statistics. An *I*^*2*^ value higher than 75% was regarded as ‘significant heterogeneity’ and a value less than 40% was considered ‘not significant heterogeneity’. Study results were considered statistically significant if the p value was below 0.05. Summary estimates were calculated by using the Mantel Haenszel random–effects model[16] in Reviewer Manager ((RevMan). Version 5.3. Copenhagen: The Nordic Cochrane Centre, The Cochrane Collaboration, 2014). This enabled an estimate of variation between studies to be made by comparing study results with a fixed effect meta-analysis result.

The calculated heterogeneity of included studies and outcome assessment precluded pooling of data for some outcomes, in which case percentage mean difference analyses of such outcomes were conducted. To facilitate comparison across studies, units of volume of antibiotic use were converted to defined daily doses per 1000 patient-days (DDD/1000 patient-days), while units for drug costs were left in the currency of the country of origin. Compliance with antibiotic guidelines was measured by percentage mean differences between intervention and control groups and length of stay was measured by differences in days between intervention and control groups.

## Results

### Search results

For this systematic review, the PRISMA statement was adopted[17] as detailed in Figure A in S1 File, PRISMA checklist highlighting study selection, which shows the results of the search and selection process. After screening 2459 studies, the removal of 237 duplicates between databases, the addition of 18 studies from bibliographies of included studies and previous systematic reviews, and the addition of 10 studies from the second updated PUBMED search, a total 378 full-text studies were reviewed. Of these, 297 studies did not meet inclusion criteria for the following reasons: were not conducted in secondary or tertiary care settings, did not answer research questions, or had inadequate study design. The characteristics and a bibliography of the 81 included studies are summarized in S1 Table, Characteristics of included studies (Table A). References for included studies (List A). Twenty-six studies assessed mortality, 25 assessed length of stay, 19 assessed volume of antibiotic usage, 16 assessed adequacy of antibiotic coverage, 15 assessed CDS uptake and use, 15 assessed cost of antibiotics, 10 assessed compliance with guidelines, and 4 assessed antimicrobial resistance. The majority of studies were conducted in the United States (45 studies).

### CDS interventions

The classification of CDS interventions by Baysari and co-workers was adopted in the present review.[9] CDS interventions found in this systematic review took four main forms: (1) stand-alone computerised decision support systems (CDSSs), (2) decision support embedded within a hospital's electronic medical record (EMR) or computerized provider order entry (CPOE) system, (3) computerized antimicrobial approval systems, and (4) antibiotic surveillance systems. Interventions were evaluated against usual care, no CDS, paper-based decision support or CDS.

### Quality of studies

This systematic review indicates that the current state of evidence for CDS in optimising antibiotic use is poor and is limited to non-Cochrane study designs as there were few randomised studies found in the literature. The majority of studies identified used before-and-after designs with very few including a control group. The included studies achieved an average score of 5.7 of a possible total of 10 on the rating scale. Random allocation of health care professionals, patients or units to a CDS intervention was rare. The majority of studies assessed an objective outcome measure (length of stay) or used a subjective measure with blinded assessment.

### Outcomes of CDS use

#### Adequacy of antibiotic coverage

Sixteen studies reported on the adequacy of antibiotic coverage. [1,5,18–31] Adequacy of antibiotic coverage was defined in individual studies and included retrospective review of antibiotic recommendations made by CDS systems and measures of prescriber compliance with published guidelines when CDS was in use. Thirteen of these contained sufficient information to be included in the meta-analysis [1,5,18,21,23–31], ten of which (1, 22, 24–27, 29–32) reported a statistically significant effect of CDS on the adequacy of antibiotic coverage. Three studies were not included in the meta-analysis since they presented insufficient data to allow pooling of outcomes. Individual and pooled estimates are shown in Figure B in S1 File, Forest plot from individual studies and meta-analysis for adequacy of antibiotic coverage.). Overall, CDS interventions were associated with an increase in adequacy of antibiotic coverage based on the random effects model [OR = 2.11, 95% CI, 1.67 to 2.66, p < 0.00001]. There was evidence of heterogeneity between studies (Chi^2^ = 55.85, df = 15, *I*^*2*^ = 73%, p < 0.00001) (Figure B in S1 File Forest plot from individual studies and meta-analysis for adequacy of antibiotic coverage. There was evidence of an effect of CDS interventions on the adequacy of antibiotic coverage for Cochrane compliant studies [OR = 1.47, 95% CI, 1.03 to 2.10, p = 0.03], and for non-Cochrane studies [OR = 2.18, 95% CI, 1.69 to 2.80, p < 0.00001] (Figure B in S1 File Forest plot from individual studies and meta-analysis for adequacy of antibiotic coverage).

#### Mortality

Twenty-six studies evaluated the impact of CDS on mortality [1,18,22,24,25,30,32–50]. Twenty studies contained sufficient information to be included in the meta-analysis [1,18,24,25,30,32–36,40–45,47–50], four of which reported a statistically significant effect of CDS on mortality. Six studies were not included in the meta-analysis because of insufficient data to allow pooling of outcomes. Individual and pooled estimates are shown in Figure C in S1 File, Forest plot from individual studies and meta-analysis for mortality. Overall, results showed that CDS interventions had a marginal statistically significant effect on mortality based on the random effects model. [OR = 0.85, 95% CI, 0.75 to 0.96, p = 0.01]. There was evidence of heterogeneity between studies (Chi^2^ = 42.37, df = 20, *I*^*2*^ = 53%, p = 0.01).

There was no evidence of an effect of CDS interventions on mortality for Cochrane compliant studies (N = 5) [OR = 0.88, 95% CI, 0.75 to 1.04, p = 0.13]. Based on non-Cochrane studies (N = 16), there was a marginal statistically significant effect of CDS interventions on mortality [OR = 0.84, 95% CI, 0.71 to 0.99, p = 0.04] (Figure C in S1 File, Forest plot from individual studies and meta-analysis for mortality.).

#### Volume of antibiotic usage

Nineteen studies reported on the impact of CDS on the volume of antibiotic usage.[20,26,32,33,36,37,39,44,46,48,50–58]. Values for total antibiotic use are summarised in Table 2. Fourteen studies showed decreases in antibiotic usage.[26,33,36,37,39,44,46,48,52,53,55–58] Two studies showed increases in antibiotic usage.[32,50] One study by Fisher and co-workers showed conflicting results as intravenous DDDs significantly decreased by 11.1% (p = 0.002), but was coupled with a compensatory increase in oral DDDs of 3.7% (p = 0.002).[20] The unit of measurement for drug use differed between studies, making it difficult to compare the impact of each intervention. A study by Burke and co-workers demonstrated an unexpected increase in DDDs which may be attributed to the declining ICU length of stay.[32] Thursky and co-workers showed a significant reduction of antibiotic DDDs (1660 to 1490 DDDs/1000 ICU bed-days), which was accompanied by a significant decrease in proportion of patients who received broad spectrum antibiotics.[26]

**Table 2:** Reduction in overall antibiotic usage with CDS interventions in secondary care.

Study	Unit of measurement	Antibiotic use in non-intervention group	Antibiotic use in intervention group	Difference	*P* value
**Agwu 2008**	Doses/day	125.8 (restricted AB) 227.5 (Unrestricted AB)	111.08 201	-11% -12%	N/A N/A
**Buising 2008**	DDD/1000 bed-days	+1.41	-0.16	-	N/A
**Burke 1999**	DDD/1000 pt-days	226	299	+32%	N/A
**Burton 1991**	DOT	8.3	7.3	-12%	0.93
**Chan 2011**	Gradient DDD/1000 pt-days	+0.916	+0.6437	-	N/A
**Cook 2011**	DDD/1000 pt-days	775.3	552.2	-28.8%	< 0.0001
**Evans 1998**	DDD/1000 pt-days	1852	1619	-13%	N/A
**Evans 1999**	DDD/1000 pt-days	1972	1882	-4.5%	N/A
**Fisher 2003**	DDD	N/A N/A	N/A N/A	-11% (IV) +3.7% (PO)	0.002 0.002
**Grayson 2004**	DDD/1000 pt-days	N/A	N/A	N/A	N/A
**Linares 2011**	Antibiotic days	6.3	2.2	-65%	< 0.001
**Mullett 2001**	Doses/patient	19.8	22	+11%	N/S
**Pestotnik 1996**	DDD/1000 pt-days	359	277	-23%	N/A
**Shojania 1998**	Antimicrobial orders/prescriber	16.7	11.3	-32%	0.04
**Sintchenko 2003**	DDD/1000 pt-days	1925	1606	-17%	0.04
**Staicu 2016**	DOT/1000 pt-days	9.5	4.4	-54%	< 0.0001
**Tafelski 2010**	Antimicrobial agents/day	1.5	1.3	-13%	0.05
**Thursky 2006**	DDD/1000 pt-days	1670	1490	-11%	N/A
**Yong 2010**	DDD/1000 pt-days	N/A	N/A	N/A	N/S

DDD, defined daily doses; DOT, duration of therapy, AB, antibiotics; N/A, not reported; N/S, not significant; PO, Oral; IV, intravenous.

#### Length of stay

Values for length of stay are summarised in Table 3. Sixteen studies showed decreases in length of stay.[24,25,30,32,34,36,37,40,41,43–45,49,52,53,59] Three studies showed increases in length of stay.[2,20,60] Three studies showed no change in length of stay.[18,50,61] Three further studies reported conflicting effects of CDS on length of stay across different intervention arms.[33,35]

**Table 3:** Length of stay associated with CDS implementation.

Study	Length of stay in non-intervention group	Length of stay in intervention group	Difference	*P* value
**Agwu 2008**	6.78 days	6.67 days	-1.62%	0.65
**Arboe 2014**	-	-	No change	N/A
**Brady 2014**	3.8 days	3.8 days	No change	N/S
**Buising 2008**	12days(pre-1) 15 days (pre-2)	15days (post-1) 13 days (post-2)	-	N/A
**Burke 1999**	10.28 days	8.84 days	-14%	N/A
**Burton 1991**	20.3 days	16 days	-21%	0.028
**Chow 2013**	9.6	8.1	-15.6	N/A
**Dean 2015**	3.1 days (baseline) 3.0days (second period)	3.0days(baseline) 2.9 days (second period)	-3.3% -3.3%	N/A
**Evans 1995**	6.2 days	5.8 days	-6.5%	N/S
**Evans 1998**	12.9days 12.9days	10 days (CDS followed) 16.7days (CDS overridden)	-22.5% +29.5%	0.001
**Evans 1999**	8.5 days	7.9 days	-7%	N/A
**Guiliano 2011**	15.7 days	17.8 days	+13%	0.58
**Fisher 2003**	N/A	N/A	+1.9%	N/A
**Kim 2013**	23 days	19.5 days	-15.2%	0.036
**King 2007**	2.8 days	2.9 days	+3.45%	0.125
**McGregor 2006**	3.99 days	3.84 days	-3.75%	0.38
**Mullett 2004**	N/A	N/A	No change	N/A
**Nachtigall 2014**	9.2 days	9.1 days (post-1) 9.9 days (post-2) 11.3 days (post-3)	-1% +7.6% +22.8%	<0.01
**Paul 2006**	9.45 days	8.83 days	-6.5%	0.055
**Pestotnik 1996**	7.5 days	7.3 days	-2.7%	N/A
**Pogue 2014**	8 days	7 days	-12.5%	< 0.001
**Rodriguez 2014**	19.5days 20.1 days	13.8 days (LRMs) 19.7 days (PMRTRs)	-29% -2%	0.156 0.943
**Rohrig 2008**	15.6 days	11.25 days	-27.9	N/A
**Sintchenko 2004**	7.15 days	6.22 days	-13%	0.02
**Thiel 2009**	28.7 days	22.4 days	-22%	0.02

LRMs, local resistance maps; PMRTRs, preliminary microbiological reports with therapeutic recommendations; N/A, not reported; N/A, not stated

#### Cost of antibiotics

Fifteen studies reported on the impact of CDS on antibiotic cost. [18,20,24,27,29,31,33,34,36,44,50,52,55,59,62] Values for cost of antibiotic use are summarised in Table 4. The unit of cost report varied making it difficult to measure the overall impact of CDS. Nine studies showed decreases in cost of antimicrobials after implementing CDS.[24,27,34,36,44,50,52,59] Four studies showed increases in cost of antibiotics following CDS implementation.[18,20,31,50] Two studies reported conflicting results on antibiotic costs.[31,33] In the study conducted by Evans and co-workers, the cost of antibiotics per patient decreased when CDS recommendations were adopted ($340 vs. $102).[33] In contrast, the cost of antibiotics per patient increased when CDS recommendations were overridden ($340 vs. $427).[33] The study by Buising and co-workers showed that CDS was superior to baseline and inferior to academic detailing in cost saving.[31] However Paul and co-workers (25) reported no difference in antibiotic costs associated with observed side effects between CDS and control groups. No study reported on the overall costs of implementation of CDS.

**Table 4:** Antibiotic cost reductions associated with CDS interventions.

Study	Unit of measurement	Antibiotic cost in non-intervention group	Antibiotic cost in intervention group	Difference	*P* value
**Agwu 2008**	N/A	N/A	N/A	-21.6%	N/A
**Arboe 2014**	N/A	N/A	N/A	Increased	N/A
**Buising 2008**	Cost of antibiotics per patient	$72.07 (baseline) $94.47(academic detailing)	$84.04	+16.6% -11.04%	N/A
**Evans 1994**	Cost of antibiotics per day	$51.93	$41.08	-21%	<0.001
**Evans 1995**	Cost of antibiotic per patient	$382.68	$295.65	-23%	N/A
**Evans 1998**	Cost of antibiotics per patient	$340	$102 (followed CDS) $427(overridden CDS)	-70% +26%	<0.001
**Evans 1999**	Average cost of antibiotics	$128	$98.06	-23.4%	< 0.004
**Fisher 2003**	N/A	N/A	N/A	+12%	N/A
**Kofoed 2009**	Total cost of antibiotics per patient in Euro	€469	€482	+2.8%	0.77
**McGregor 2006**	Total cost of antimicrobials	$370,006	$285,812	-23%	N/A
**Mullett 2004**	Cost of antibiotic per patient	$274.79	$289.60	+5%	NS
**Paul 2006**	Total cost of antibiotic in Euro	€623.2	€565.4	-9%	0.007
**Pestotnik 1996**	Antibiotic cost per patient	$122.66	$51.90	-58%	-
**Potasman 2012**	Total antibiotic expenditure	4.1 million NIS	3.4 million NIS	-17%	N/A
**Shojania 1998**	Annual cost of antibiotics	N/A	N/A	$90,000/year	N/A

N/A not reported, N/S not significant, NIS = New Israeli Shekel

#### Compliance with guidelines

Ten studies reported on the impact of CDS on compliance with guidelines.[19,28,31,35,51,60,63–66] Values for the percentage of compliance with guidelines are summarised in Table 5. CDS effects were measured as absolute percentage differences between CDS and intervention groups. All studies demonstrated that CDS improved adherence to guidelines (see Table 5). Guiliano and co-workers showed that CDS improved adherence to sepsis resuscitation and management bundles.[60] Tafelski and co-workers showed that ICU mortality was significantly increased in low adherence group (LAG) compared to high adherence group (HAG) (OR = 2.43, 95% CI 1.126 to 5.243).[39]

**Table 5:** Compliance associated with CDS.

Study	Compliance in non-intervention group	Compliance in intervention group	Difference	*P* value
**Buising 2008**	65% (baseline) 75% (academic detailing)	85% (CDS) 85% (CDS); OR = 1.99 [1.07, 3.69], p = 0.02).	+20% +10%	0.05 0.05
**Demonchy 2014**	26.5% 34%	32% (post DS) 43.5% (post CDS)	+5.5% +9.5%	N/A
**Guiliano 2011**	57.6% (resuscitation bundles) 84.5% (management bundles)	68.2% 86.8%	+10.6 ++2.3%	0.003 0.48
**Karsies 2014**	15%	76%	+61%	
**Nachtigall 2014**	61.4%	92% (post-1) 76.3% (post-2) 71.1% (post-3)	+30.6% +14.9% +9.7%	<0.001 <0.001 -0.001
**Revolinski 2015**	69.7%	71.2%	+1.5%	0.605
**Tafelski 2010**	39.8%	90.8%	51%	<0.05
**Van Sise 2012**	85.7%	92.6%	+6.9%	<0.005
**Westphal 2011**	49%	67%	+18%	<0.001
**Grayson 2004**	N/A	76%	N/A	N/A

N/A, not reported; CDS, clinical decision support

#### Antimicrobial resistance

Four studies reported on AMR.[36,46,48,54] In a study by Chan and co-workers, the rate of methicillin resistant *Staphylococcus aureus* (MRSA) decreased from 65–70% before the implementation of the antimicrobial approval system in 2003 to less than 60% in 2009.[46] Buising and co-workers showed a trend after the introduction of an antimicrobial approval system towards increased susceptibility of *S*. *aureus* to methicillin and increasing susceptibility of *P*seudomonas spp. isolates to both carbapenems and aminoglycosides.[48]

#### Use and implementation

Fifteen studies assessed aspects of the use and implementation of CDS, such as user satisfaction,[2,33,52,67–69] user uptake,[37,48,59,68] and acceptance of CDS recommendations.[38,46,50,51,59,70,71,72] Six studies showed improvement of user uptake and satisfaction. In a study by Chow and co-workers, the proportion of times when CDS was used when antibiotics were prescribed increased from 23% in phase (1) to 38% in phase (2) and to 87% in phase (3).[70] CDS recommendations were accepted in 40% to 89% of cases. Buising and co-workers showed that the use of an approval system increased between 2005 and 2006 and reached a plateau of 250–300 new approvals per month.[48]. Stevenson and colleagues showed that agreement with CDS recommendations had a pooled odds ratio (1.88, 95% CI, 1.01–3.56, p = 0.04).[38] In contrast, six studies showed poor user uptake of CDS recommendations. In a study by Hum and co-workers, 37% of those eligible used CDS while working in a Neonatal Intensive Care Unit.[68] Sintchenko and co-workers [73] showed a low level of CDS adoption as only one third of CDS recommendations were accepted, while Evans and co-workers showed that 37% of CDS recommendations were accepted.

## Discussion

### Main findings

Evidence for the impact of CDS on antibiotic use in hospital inpatient settings has been reviewed systematically. Almost half of the studies included in the present systematic review did not appear in previous systematic reviews. This highlights the pace of the introduction and evaluation of health information technology in hospital settings. Therefore, this systematic review extends previous evidence, including studies never evaluated previously.

Studies were extremely variable in the types of CDS interventions and in the outcomes assessed. The most commonly assessed outcomes were mortality, length of stay, volume of antibiotic use and adequacy of antibiotic coverage. Other outcomes assessed included system uptake, antimicrobial resistance, cost and compliance with guidelines. Only a small number of studies of this systematic review assessed health outcomes (mortality and adequacy of antibiotic coverage) which may limit the strength of evidence needed to reflect on CDS design, selection and implementation.

The principal findings of the meta-analysis indicate evidence that some studies of CDS interventions were associated with improvements in adequacy of antibiotic coverage (by more than 100%) and patient mortality (reducing the risk of death by about 15%). However, these findings were likely to be driven by data from poor quality studies. Increases in compliance with guidelines have been noted in the present review. Drawing conclusions about the effects of CDS on length of stay and cost of antibiotics is difficult since results from the present review are conflicting. A meta-analysis by Baysari and co-workers (9) showed similar findings of the impact of CDS interventions on adequacy of antibiotic coverage The current systematic review indicated conflicting results on CDS uptake as some studies showed improved uptake while other showed poor adoption. A study by Demonchy and coworkers[19], has highlighted uptake and implementation issues of CDS as a major barrier. The impact of CDS interventions would have been greater if used regularly by prescribers.

### Strengths

This systematic review provides a comprehensive, up-to-date overview of CDS interventions aimed at optimising antibiotic use in the hospital inpatient setting. A wide range of outcome measures was assessed including outcomes that have not been previously evaluated, such as cost, system uptake and antimicrobial resistance. It is noteworthy that non-randomised designs have been commonly utilised in evaluations of health informatics developments, as evidenced by this systematic review. The present review included studies that have not been included in other systematic reviews.

Given that the quality and study design of included studies were generally poor and the heterogeneity in respect of study quality and end points, the synthesis of the studies was problematic. However, it was possible to conduct meta-analysis and subgroup analysis which adds to the strength of this review.

### Limitations and future research

The present systematic review is limited by the quality of studies included for analysis coupled with limitations inherent in the applied methods. All studies were eligible for inclusion in the meta-analysis but information contained in studies enabled meta-analysis to be conducted for two outcomes: adequacy of antibiotic coverage (n = 13 studies) and mortality (n = 20 studies). The number of studies that reported other outcome measures (e.g. volume of antibiotic use and cost) in a uniform way was not sufficient for other meta-analysis to be conducted.

Heterogeneity in study designs, CDS interventions, outcomes, implementation and contextual factors make it difficult to reach firm conclusions about the impact of CDS. Subgroup analysis was not successful in explaining or even reducing heterogeneity across subgroups. This indicates that heterogeneity was inherent in poor methodological and intervention designs.

There is a possibility that selective reporting may reduce the validity of some of the conclusions. A marginal reduction of mortality is a key finding of from this systematic review; however, this finding is based on a limited number of studies (n = 20). Selective reporting could not be controlled, as it is not clear how many studies that might have found an increase in mortality it would take to nullify or even reverse the findings here. Therefore, an assurance that there is no risk of an increase in mortality is not possible. Caution needs to be applied with regards to the possibility of publication bias or evaluation by developers. It should be clear that an external evaluation should be reported using accepted mixed methods research. There is a lack of literature about the impact of CPOE without explicit CDS from commercial vendors: this may be due to publication bias.

Future work should include conducting high quality systematic multi-site comparative studies of different CDS interventions for antibiotic prescribing. More qualitative work is required to highlight the barriers and facilitators of adopting CDS technology and better understanding users’ perceptions and attitudes towards CDS interventions to trigger high adoption and uptake by providers.

## Conclusion

This review indicates that CDS interventions can be effective in optimising antibiotic use in hospitals. The findings of this review can be used to enrich the debate around the impact of CDS on antibiotic optimisation. This review demonstrates the efficacy of CDS in optimising the adequacy of antibiotic coverage across different settings. However, evidence on the effect of CDS on clinical outcomes, economic outcomes and volume of antibiotic use was limited. CDS appears to be safe because the present review has not shown any significant risks such as worsening mortality or length of stay. CDS presents a promising future for optimising antibiotic use and improving patient care. However, in order to reach firm conclusions about the impact of CDS on antibiotic use, more high quality studies are needed within different settings and in different health systems.

## Supporting information

S1 File PRISMA checklist highlighting study selection (Figure A). Forest plot from individual studies and meta-analysis for adequacy of antibiotic coverage (Figure B). Forest plot from individual studies and meta-analysis for mortality (Figure C).
(DOCX)

S1 Table Characteristics of included studies (Table A). References for included studies (List A).
(DOCX)

S1 Appendix PRISMA Search strategy details. (DOCX)

S1 Checklist PRISMA 2009 checklist. (DOC)
