# Supplementary material for: The effectiveness of computerised decision support on antibiotic use in hospitals: A systematic review
Source: PLoS One. 2017 Aug 24;12(8):e0183062. doi: 10.1371/journal.pone.0183062 (PMC5570266; doi:10.1371/journal.pone.0183062)
Supplement: S1 File — PRISMA checklist highlighting study selection (Figure A). Forest plot from individual studies and meta-analysis for adequacy of antibiotic coverage (Figure B). Forest plot from individual studies and meta-analysis for mortality (Figure C). (DOCX) [file pone.0183062.s001.docx]

**Figure A: PRISMA checklist highlighting study selection.**

**MEDLINE = 509**

**PubMed = 747**

**CINAHL = 96**

**Cochrane = 80**

**HMIC = 18**

**PsychINFO = 52**

**Web-Science = 157**

**EMBASE = 800**

## **Identification**

**Duplicates removed
N = 237**

**Records screened**

**N = 2459**

**Screening**

**Title and abstracts screening**

**N = 2222**

**Records excluded**

**N = 1872**

**Non-duplicate studies of previous systematic reviews**

**N = 18**

**Eligibility**

**Full-text articles assessed for eligibility
N = 350+ 18+10 = 378**

**Second search by PUBMED (2014-2016)**

**N = 10**

sscond

**Records excluded**

**N = 297**

**Primary care, ambulatory, outpatient settings.**

**Did not answer research question.**

**Inappropriate study design (Qualitative studies, editorials, case reports, and case series).**

**Studies included in qualitative synthesis**

**N = 81**

## **Included**

**Figure B: Forest plot from individual studies and meta-analysis for adequacy of antibiotic coverage**


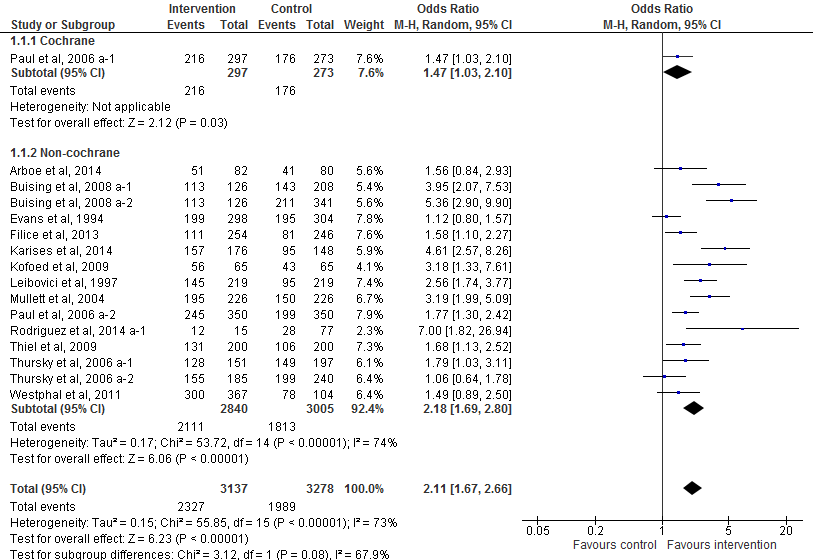


**Figure C: Forest plot from individual studies and meta-analysis for mortality**

***
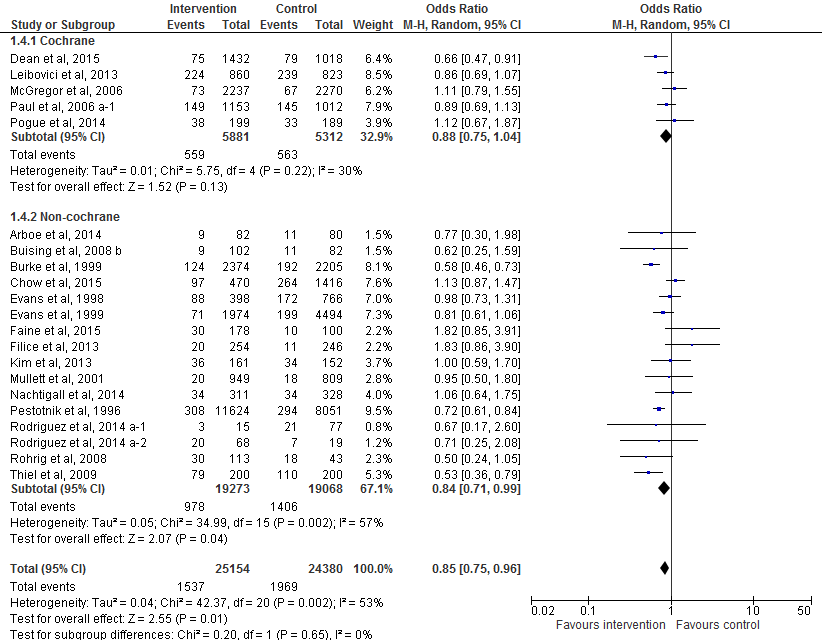
***
