# Supplementary material for: The effectiveness of computerised decision support on antibiotic use in hospitals: A systematic review
Source: PLoS One. 2017 Aug 24;12(8):e0183062. doi: 10.1371/journal.pone.0183062 (PMC5570266; doi:10.1371/journal.pone.0183062)
Supplement: S1 Table — Characteristics of included studies (Table A). References for included studies (List A). (DOCX) [file pone.0183062.s002.docx]

| S1 Table  Table A: Characteristics of included studies | | | | | | | |
| --- | --- | --- | --- | --- | --- | --- | --- |
| Author, year | Quality | Setting, Country | Participants | Study design | Intervention | Primary outcome | Main findings |
| Agwu et al, 2008[1] | 8/10 | 175-bed tertiary children hospital, USA | 199 participants | Single-site, uncontrolled before and after | Approval system | Delivery times for restricted and unrestricted antibiotics, number of dispensed restricted & unrestricted antibiotics, cost, patient complexity, length of stay | No difference in dispensing time for restricted antibiotics, but time was reduced for unrestricted antibiotics (p<0.001). Cost of restricted antibiotics decreased by 21.6% (no p-value reported) but cost of unrestricted antibiotics did not change. Number of requests for approval increased post system (220 vs. 342 requests per month, no p-value reported). Number of doses of restricted antibiotics dispensed decreased by 11%. No difference in complexity or length of stay. |
| Arboe et al, 2014 [2] | 4/10 | Acute care hospital, Denmark | 511 patients | Single-site, uncontrolled before and after | CDSS | Adequacy of antibiotic coverage, cost, length of stay and mortality | Antimicrobial coverage rates in the retrospective group for TREAT, physicians and guidelines were 65%, 51%, and 79%, respectively, and 68%, 62%, and 77%, respectively, for the prospective group; TREAT provided a significant lower coverage than local guidelines (p ˂ 0.001); no differences were found in the length of stay, or hospital or 30-day mortality. |
| Bourdeaux et al, 2014 [3] | 4/10 | Mixed medical and surgical tertiary ICU, UK | N/A | Single-site, uncontrolled before and after | DS within CPOE/EHR | Proportion of eligible patients prescribed chlorhexidine | Proportion of eligible patients prescribed chlorhexidine increased from 55.3% to 90.4% after DS (p˂0.001). |
| Brady et al, 2014 [4] | 5/10 | Children hospital, USA | 31 patients | Single-site ITS | DS within CPOE/EHR | Discharge on oral therapy | Increased percentage of children with osteomyelitis discharged on oral therapy from no cases (0%) to 9 cases (100%); differences in length of stay and cost were not significant. |
| Buising et al, 2008a [5] | 6/10 | Teaching hospital, Australia | 740 patients | Single-site, uncontrolled before-and-after,  ITS | CDSS | Compliance and adequacy of empirical antibiotic prescribing | The odd ratio for concordant therapy in the academic detailing period compared to the baseline period was (OR = 2.79 [1.88, 4.14], p ˂ 0.01); the odd ratio for concordant therapy in the computerised decision support period compared to the academic detailing period was (OR = 1.99 [1.07, 3.69], p = 0.02). |
| Buising et al, 2008b [6] | 5/10 | 365-bed tertiary referral hospital, Australia | Patients prescribed broad spectrum antibiotics | Single-site, uncontrolled before and after | Approval system | Uptake of tool, antibiotic consumption, antibiotic resistance, mortality, length of stay | Approval system uptake increased and plateaued at 250-300 new approvals per month. A fall in use of 3rd and 4th generation cephalosporins occurred (p<0.01). Patterns of resistance of common pathogens remained stable. No change in mortality rate or length of stay. |
| Burke et al, 1999 [7] | 5/10 | Teaching hospital, USA | 11,634 patients | Single-site, uncontrolled before-and-after | CDSS | DDD/1000 patient-days, mortality, length of stay, adverse drug rate, and antimicrobial resistance patterns | Increased DDD/1000 patient-days from 226 to 299; declining ICU length of stay from 3.6 days to 2.8 days; decreased overall length of stay; decreased mortality and the incidence of adverse drug events. |
| Burton et al, 1991 [8] | 8/10 | Tertiary care medical centre, USA | 147 patients | Single-site RCT | CDSS | Response rate, incidence of toxicity, means length of stay, length of aminoglycoside therapy, and cost | Intervention group had higher response rates (60% vs 48%); insignificant decrease in the incidence of toxicity was seen (from 9.7% to 5.1%); significant decrease in the duration of hospital stay in (from 20.3 to 16 days, p= 0.028); potential cost savings of $ 1311 per patient can be achieved. |
| Caplinger et al, 2016 [9] | 5/10 | Teaching hospital with surgical and medical intensive care units, USA | 68 patients before and 59 patients after | Single-site uncontrolled before and after | CDSS | Antipseudomonal carbapenems (APC) initiations per1000 patient-days | Aggregate monthly APC initiations decreased from 7.01 to 6.14 per 1000 patient-days after the implementation (p=0.03). Post- intervention APC initiations for patients with low-risk β-lactam histories decreased from 92% to 83% (p=0.17). No adverse events were observed in patients with low-risk β-lactam histories. The intervention was associated with a reduction in APC initiations. |
| Chan et al, 2011a [10] | 5/10 | ICUs of a hospital, Taiwan | 762 patients (465 before and 297after) | Single-site, uncontrolled before and after | DS within CPOE/EHR | The frequency of cases with undesirable peak (˂4µg/ml) and trough (≥2µg/ml) serum gentamicin concentration and patient satisfaction | The frequency of gentamicin regimens that resulted in undesired levels decreased (13.5%) after implementation of the gentamicin online calculator compared to before the implementation (32.7%). The users expressed satisfaction with the dosage calculator (mean score, 4.9; n=18-20). |
| Chan et al, 2011b  [11] | 6/10 | 3500-bed medical centre, with 400 ICU beds, Taiwan | Patients for which restricted and non-restricted antibiotics were prescribed | Single-site, uncontrolled ITS | Approval system | Use of restricted and non restricted antibiotics, hospital infection rates, mortality rates, incidence of C. diff infection, resistance profile | Use of 3rd and 4th generation cephalosporins, fluroquinolones & glycopeptides reduced following deployment (p<0.001) Use of carbapenems increased, as did some front line antibiotics (p<0.001). No change in hospital infection rates or mortality rates. Mixed results for resistance profiles. No change in rate of C diff. |
| Chow et al, 2015 [12] | 6/10 | 1500-bed tertiary-care hospital, Singapore | 1886 patients who were prescribed piperacillin-tazobactam or carbapenem for empiric therapy | Single-site, prospective observational cohort | CDSS | The extent to which hospitalised patients received antibiotics recommended by CDSS, 30-day all-cause mortality, the incidence of C. diff infection (CDI) and multidrug resistant organism (MDRO). | One-quarter of the 1886 patients received CDSS-recommended antibiotics. More patients treated for pneumonia (33.2%) than sepsis (12.2%) and urinary tract infections (7.1%) received CDSS-recommended antibiotic therapies. Receipt of antibiotics according to CDSS’s recommendations lowered mortality risk of patients (OR 0.54, 95% CI 0.26-1.10, p=0.09). No effect was seen on the incidence of C. diff (OR 1.02, 95% CI 0.34-3.01), and multidrug resistant organism (OR 1.06, 95% CI 0.42-2.71). |
| Chow et al, 2015[12] | 7/10 | 1500-bed tertiary care teaching centre, Singapore | All patients with electronic prescriptions of antibiotics | Single-site, three-phase cohort study | CDSS | Trend of completed launches for guidance and via auto-trigger per month, trend of proportion of accepted CDSS recommendations per month, factors associated with acceptance of CDSS recommendations | In phase 1, 23% of CDSS launches were completed which rose to 38% in phase 2 and then to 87% in phase 3.  Amongst completed launches for guidance, 89% of CDSS recommendations were accepted versus 40% amongst completed launches via auto-trigger. Amongst CDSS launches for guidance, being from a medical department [adjusted odds ratio (aOR)= 1.20, 95% confidence interval (CI) 1.04-1.37] and CDSS launches during on-call (aOR=1.81, 95% CI 1.61-2.05) were independently associated with acceptance of CDSS recommendations |
| Cook et al, 2011[13] | 6/10 | 861-bed tertiary care teaching hospital, USA | Patients for which MRSA and C. diff culture reports were queried | Single-site, uncontrolled before and after | DS within CPOE/EHR | Use of antibiotics incidence of nosocomial infection with C. diff and MRSA, pharmacy interventions and accepted recommendations | There was a 98.1% increase in the number of antibiotic recommendations made (p<0.0001). There was a 28.8% decrease in use of antibiotics following intervention (p<0.0001). MRSA infections decreased by 45.2% (p<0.0001) and a18.7% decrease in C. diff but this was non-significant. |
| Cox et al, 2011 [14] | 8/10 | 593-bed tertiary care teaching medical centre, USA | 216 patients | Single-site, uncontrolled before and after (historical control) | DS within CPOE/EHR | Appropriate aminoglycoside dosing, number of aminoglycoside orders, and peak and trough concentrations | Significant increase in adherence with reference standards (41% to 80%, p < 0.001); significant increase in appropriate selection of correct initial interval (63% to 87%, p < 0.001); significant increase of goal trough concentrations (59% to 89%, p < 0.004). |
| Dean et al, 2015 [15] | 7/10 | 7 Intermountain Healthcare hospital EDs in Utah urban corridor, USA | 4758 patients (2071 usual care group vs 2687 intervention group) | Multisite, controlled before and after | CDSS | 30-day, all-cause mortality and patient disposition from the ED | There was no difference overall in severity-adjusted mortality between intervention and usual care EDs post-tool deployment (odds ratio OR=0.69; 95% CI 0.41 to 1.16). Patients with community acquired pneumonia experienced significantly lower mortality (OR=0.53; 95% CI 0.28 to 0.99), whereas mortality was unchanged among patients with health care-associated pneumonia (OR=1.12; 95% CI 0.45 to 2.8). Patient disposition from the ED post deployment adhered more to tool recommendations. |
| Demonchy et al, 2014 [16] | 5/10 | Emergency departments of three teaching centres, France | 912 patients | Multisite, uncontrolled before-and-after (3 study periods after intervention) | CDSS and DS within CPOE/EHR | Compliance of antibiotics prescribed with national guidelines | Compliance of prescriptions to guidelines was improved following the use of CDSS in one ED (absolute increase +20%, p = 0.007); the choice of antibiotic was improved following the use of CDSS [OR = 1.94 (95% CI 1.13-3.32)]. |
| Devabhakthuni et al, 2012 [17] | 6/10 | Medical centre, USA | Patients receiving vancomycin | Single-site, uncontrolled before and after | DS within CPOE/EHR | Appropriate initial vancomycin regimens, appropriate initial trough concentrations, appropriate time of initial trough levels, appropriate number of doses administered prior to trough levels, number of levels drawn, duration of therapy | There was an increase in appropriate vancomycin doses from 40% to 56% (p<0.001) but no change in the proportion with an appropriate dosing interval. The initial trough concentration was higher before the intervention than after (17mcg/ml vs. 13 mcg/ml; p=0.048). |
| Diasinos et al, 2014 [18] | 5/10 | 320-bed teaching hospital, Australia | Patients receiving gentamicin | Single-site, retrospective audit and interviews | DS within CPOE/EHR | Compliance of gentamicin prescribing (empirical cases, STAT doses and continued cases) with the Australian Therapeutic Guidelines, version 14 (2010). To determine why resources were effective or ineffective in achieving compliance to guidelines | Intravenous gentamicin was used in 545 cases, 81% of which were for short-term therapy (≤ 48 h). Of the continued dosing cases, 55% went unmonitored and the computerised dose recommendation service was rarely used. 91.4% (498/545) of subsequent empirical dosing, 72.8% (206/283) of empirical dosing interval and 44.1% (30/68) of therapeutic drug monitoring (TDM) requests were compliant with therapeutic guidelines. |
| Evans et al, 1990 [19] | 8/10 | 520-bed tertiary private hospital, USA | Surgical patients receiving antibiotic prophylaxis | Single-site, uncontrolled before and after | Surveillance system | Cost of antibiotics, antibiotic usage | Cost of antibiotics per surgical patient decreased from $170 to $162 (No p-value reported). Patients received fewer doses of antibiotics following surveillance system (19 vs. 13, p<0.001). 10% of patients were still receiving antibiotics 7-days post-op pre surveillance system, compared with 5% post surveillance system (p<0.001). |
| Evans et al, 1994 [20] | 4/10 | 520-bed tertiary private hospital, USA | House staff physicians prescribing antibiotics | Single-site, cross-over design | CDSS | Adequacy of antibiotic coverage, timeliness and users’ perceptions | Significant increase in adequacy of antibiotic coverage (from 77% to 94%, p < 0.001); significant earlier prescribing of antibiotic (from 21 h to 12 h, p <0.035); 88% of users recommended Antibiotic Consultant CDS and felt that it improved patient care. |
| Evans et al, 1995 [21] | 4/10 | 12-bed shock/trauma/ respiratory ICU, USA | 636 patients | Single-site, uncontrolled before-and-after | CDSS | Adoption rates, adequacy of antibiotic coverage, cost, and adverse drug events | Recommendations of Antibiotic Assistant were adopted 218 (37%) times; cost of antibiotics decreased (from $61.72 to $50.97); insignificant decrease of adverse drug events (from 2.4% to 0.09%, p = 0.164). |
| Evans et al, 1998 [22] | 6/10 | 12-bed ICU of 520-bed tertiary private hospital, USA | 1681 patients | Single-site, uncontrolled before-and-after | CDSS | DDD/100 occupied bed-days, cost of hospitalisation, and cost of surveillance of adverse drug events | Significant reductions were seen in DDD/100 occupied bed-days (p ˂ 0.001); significant reductions in cost of hospitalisation (p ˂ 0.001) and in cost of antimicrobial agents (p ˂ 0.001). |
| Evans et al, 1999 [23] | 8/10 | 520-bed tertiary private hospital, USA | Surgical patients receiving excessive antibiotics prophylaxis (4494 patients before and 1974 patients after) | Single-site, uncontrolled before and after | Surveillance system | Number of patients receiving excessive dosages of antibiotics, rate of adverse drug events | During the post period, 44% of patients received excessive dosages, compared to 50% in the pre period (p<0.001). Patients received excessive dosages for 2.9 days during post period and 4.7 days during the pre (p<0.001). Fewer doses of antibiotics were given post intervention, and fewer grams at less cost. 0.3% adverse drug evens were found post, compared to 0.9% pre (p<0.001). |
| Faine et al, 2015 [24] | 7/10 | ED of teaching Level 1 trauma centre, USA | 278 patients (100 pre- and 178 post-intervention) | Single-site, uncontrolled before-and-after | CDSS | The proportion of appropriate vancomycin doses based on actual body weight, mortality and length of stay | The dose calculation tool was associated with an increase in mean vancomycin dose ([14.1±5.0] vs. [16.5±5.7] mg/kg, p<0.001) and a 10.3% absolute improvement in first-dose appropriateness (34.4% vs. 24.0%, p=0.07). 28-day mortality (odds ration OR1.72; 95% CI [0.76-3.88], p0.12) was not affected. |
| Filice et al, 2013 [25] | 5/10 | Teaching veterans hospital, USA | 500 patients | Single-site cohort study | CDSS | Appropriateness of antibiotic coverage, and mortality | CDSS courses were more likely to be appropriate (111/254, 44%) compared with non-CDSS (81/246, 33%, p = 0.013); CDSS courses were more likely to be appropriate than non-CDSS courses (OR= 1.83, CI, [1.13-2.98]; mortality was not significantly correlated with CDSS use (OR, 1.5; 95% CI, 0.6-3.5). |
| Fischer et al, 2003 [26] | 5/10 | Teaching hospital, USA | Hospitalised patients receiving targeted medications | Single-site, uncontrolled before-and-after | CDSS | DDD, length of stay, case-mix index and total drug cost | Significant decrease of the intravenous DDD by 11.1% (p=0.002) and significant increase of the average oral DDD by 3.7% (p = 0.002); the average hospital length of stay and case-mix index subtly increased, while total drug cost increased by 12%. |
| Garner et al, 2015 [27] | 5/10 | 38- bed NICU of teaching hospital, USA | 79 patients (34 patients pre- and 45 patients post-intervention) | Single-site, uncontrolled before-and-after study | DS within CPOE/EHR | Overall, prescribing and omission error rate per order | The overall error rate per order decreased from 1.7 to 0.8 (p<0.001) and potential error rate from 1.0 to 0.06 (p<0.001). The reduction in omission rate per order from 0.2 to 0.1 was not significant (p=0.174). The prescribing error rate per order increased from 0.4 to 0.7 (p=0.3). |
| Giuliano et al, 2011 [28] | 4/10 | 2 adult ICUs, USA | 135 patients | Multisite, uncontrolled before-and-after | CDSS | Adherence to the resuscitation and management sepsis bundles, and time to complete these two bundles and time to antibiotic administration | Significant improvement of adherence to resuscitation sepsis bundle (p = 0.01) and significant decreased time to administer antibiotics (p = 0.006); no significant improvement was seen for adherence to management bundle or time to complete the resuscitation or management bundles. |
| Grayson et al, 2004 [29] | 4/10 | 430-bed tertiary teaching hospital, Australia | Patients prescribed ceftriaxone, cefotaxime and vancomycin for which approval was sought | Single-site, Uncontrolled before and after | Approval system | Number of approved/non approved courses of ceftriaxone/ cefotaxime and vancomycin, concordance between system recommendation and use | No change in use of antibiotics. 48% of phone approvals were substituted by system approvals |
| Hall et al, 2015[30] | 8/10 | ED in a tertiary care hospital, USA | 597 patients (220 in the pre-CPOE and 377 in the post-CPOE group) | Single-site, uncontrolled before and after | DS within CPOE/EHR | Rate of appropriate initial ED vancomycin doses, as per hospital protocol. | Appropriate dosing of vancomycin increased by 21.9% (45.5% to 67.4%, p˂ 0.05). In critically ill patients, there was 16.3% increase in appropriate dosing with 44.7% (38/85) in the post-CPOE group compared with 28.4% (19/67) in the pre-CPOE group. |
| Hamad et al, 2015[31] | 8/10 | 950-bed acute NHS teaching hospital, UK | Patients receiving vancomycin and gentamicin | Single-site, uncontrolled before and after | DS within CPOE/EHR | The accuracy of vancomycin and gentamicin initial doses | Gentamicin dose errors fell from 61.5% to 44.2%, p˂0.01. Incorrect vancomycin loading doses fell from 58.1% to 32.4%. Incorrect vancomycin first maintenance doses fell from 55.5% to 33.1%, p˂0.01. Loading and first maintenance vancomycin doses were both incorrect in 37.4% of patients before and 13.4% after calculator implementation, p˂0.01. |
| Haynes et al, 2011  [32] | 6/10 | 705-bed tertiary hospital for intervention and 327-bed tertiary hospital for control, USA | Eligible patients received antimicrobial surgical prophylaxis | Multisite, controlled before and after | DS within CPOE/EHR | The percentage of surgeries with timely discontinuation of antibacterial surgical prophylaxis after surgery, and post-surgical infection rate | Significant increase in timely discontinuation of antibacterial agents in the intervention group from 38.8% to 55.7% (p < 0.001); the prevalence of infection was 14% after the intervention implementation. |
| Heininger et al, 1999 [33] | 4/10 | Teaching hospital, USA | 447 patients | Single-site, retrospective study | CDSS | Appropriateness of diagnosis, drug-bug match, and therapeutic choice of antibiotic | 74% of the empirically prescribed antibiotics matched the antibiotic susceptibility patterns; similarly, 90% of the calculated therapy corresponded with the antibiograms. |
| Helmons et al, 2010 [34] | 5/10 | 700-bed teaching hospital, The Netherlands | 1788 patients admitted to the ICU | Single-site, retrospective study | CDSS | Dose adjustments, duration of exposure and associated costs | Dose adjustment of antimicrobials was omitted in 163 patients (86%) with moderate renal failure and 13 patients (54%) with severe renal failure. Excessive exposure was most frequently detected in patient receiving fluconazole and ciprofloxacin (median duration of 6 days). In one ICU, more than €16,000 can be saved annually by adjusting the dosage according to renal function of frequently prescribed antimicrobials. |
| Hermsen et al, 2012 [35] | 7/10 | 624-bed acute care medical centre, USA | 607 patients before and 791 patients after | Single-site, uncontrolled before and after | Surveillance system | Alerts generated, actionable alerts, proportion of alerts resulting in an intervention, proportion of recommendations accepted. | Implementation led to an increase in the number of intervention attempts (pre = no interventions documented, post = 284), but only 30% of alerts led to interventions. 88% of interventions were accepted. |
| Hulgan et al, 2004 [36] | 4/10 | Teaching hospital, USA | Patients for which quinolone orders, oral or intravenous, were placed. | Single-site, uncontrolled ITS | DS within CPOE/EHR | The proportion of inpatient quinolone orders placed for oral formulations before and after deployment of the intervention | There was an increment of oral quinolone orders from 4202 (56%) before the intervention to 4760 (62%) after; the time series analysis showed a significant overall 5.65% increase (95% CI 2.8-8.4%; p ˂ 0.001) in weekly oral quinolone orders after deployment of the intervention. |
| Hum et al, 2014[37] | 5/10 | Two NICUs affiliated with an teaching hospital, USA | 452 patients who were prescribed antibiotics | Multi-centre prospective cohort and user survey | CDSS | Patterns of use of CDSS and users’ acceptance and satisfaction | 1,303 CDSS activations for 452 patients occurred representing 22% of patients prescribed antibiotics during this period. Most survey respondents (63%) were aware of the CDSS tool, but fewer (37%) used it during their most recent NICU rotation. Summarizing culture results (43%) and the provision of antibiotic recommendations (48%) were considered the most useful features of CDSS. |
| Hwang et al, 2004 [38] | 6/10 | 600-bed teaching hospital, Taiwan | 121 patients | Single-site, post-intervention with control | CDSS | Peak and trough levels of gentamicin | Peak levels were lower in the control group than in the intervention group (6.4 mg/l vs 6.13 mg/l, p=0.035) and trough concentrations were lower in the intervention group than control (0.89 mg/l vs. 1.06 mg/l, p˂0.001). |
| Karsies et al, 2014 [39] | 6/10 | Tertiary care PICU, USA | Chart review of patients were reviewed one year before and after DS | Single-site, uncontrolled before and after | DS within CPOE/EHR | Risk-appropriate antibiotics, culture-appropriate antibiotics, time from culture to appropriate antibiotics | Post intervention patients were more likely to receive risk-appropriate antibiotics (15% vs. 76%, p<0.0001) and culture appropriate antibiotics (64% vs. 89%, p<0.0001). Overall, no difference in time to first antibiotics between groups (p=0.99) but post patients had a shorter time from culture results to appropriate antibiotics, compared to pre patients (5.9 vs. 9.6 h, p<0.0001). |
| Kazemi et al, 2011 [40] | 4/10 | Neonatal ward of 400-bed tertiary teaching hospital, Iran | Patients with antibiotic prescriptions were reviewed to identify prescribing errors | Single-site, uncontrolled before and after (three periods) | DS within CPOE/EHR | Proportion of medication errors (prescription and transcription errors) | Rate of non-intercepted errors fell from 53% during the pre-intervention period to 34% following the introduction of DS (p<0.001). No change in rate of errors was demonstrated with CPOE. |
| Kim et al, 2013 [41] | 8/10 | 1950-bed tertiary care university hospital, South Korea | Medical review of patients were reviewed one year before and after DS implementation | Single-site, uncontrolled before and after | DS within CPOE/EHR | Time between culture and administration of appropriate antibiotics, length of stay and 30-day mortality | Appropriate therapy was started earlier in the intervention group compared to control (13.5 vs.20, p=0.136). Median length of stay decreased from 23 to 19.5 days (0.036). No change in mortality. |
| King et al, 2007 [42] | 5/10 | Tertiary paediatric hospital, Canada | 334 patients | Single-site, uncontrolled before-and-after | DS within CPOE/EHR | The frequency of ordering of antibiotics; length of hospital stay; disease severity; trainees’ perceptions | Significant reduction of patients receiving antibiotic from 35% to 22% following the introduction of a Clinical Evidence Module (CEM) (relative decrease 37%, p = 0.016). |
| Kofoed et al, 2009 [43] | 6/10 | 800-bed university hospital, Denmark | 161 patients | Single-site, non-interventional cohort | CDSS | Adequacy of antibiotic coverage for empirical treatment, types of antimicrobials used and cost | Adequacy of antibiotic coverage was significantly higher by CDSS than that by clinical practice (86% vs 66%, p =0.007). There was no significant difference in the cost of future resistance between treatments chosen by CDSS and those by physicians. There was no significant difference in the direct cost for antibiotics while there were higher costs with CDSS when including patients without antibiotic therapy. There was a significant lower cost of side effects with CDSS. |
| Kweekel et al, 2004 [44] | 6/10 | University hospital, Netherlands | Patients who were prescribed restricted antibiotics,  . | Single-site, uncontrolled before and after | DS within CPOE/EHR | Appropriate antibiotic prescribing | Percentage of correct antibiotics increased from 25% to 43.7% after implementation of DS (p<0.001). Errors in duration of antibiotics therapy decreased from 22% to 9.7% (p=0.01). |
| Larsen et al, 1989 [45] | 6/10 | Teaching hospital, USA | 6831 patients | Single-site, uncontrolled before-and-after | DS within CPOE/EHR | Timing of antimicrobial prophylaxis, post-operative infections, proportion of pre-operative antimicrobial use | Significant improvement in rates of postoperative wound infectious complications (p ˂ 0.03); significant improvement in the timeliness of preoperative antimicrobial prophylaxis (p ˂ 0.001); no significant effect on the frequency of antimicrobial use per patient on the day of surgery (pre-intervention 79%, post-intervention 82%). |
| Leibovici et al, 1997 [46] | 5/10 | Teaching hospital, Israel | 496 patients | Single-site, non-interventional comparative cohort | CDSS | Cohort: The percentage of appropriate empirical antibiotic treatments, | The recommendations of CDSS were significantly inappropriate in 50 patients (23%, p ˂ 0.05) and superfluous (11%) compared to physicians’ recommendations in 91 patients where it was inappropriate in 42% and superfluous in 15% of the patients. |
| Leibovici et al, 2013[47] | 8/10 | Teaching hospital, Israel | 1683 patients | Cluster RCT | CDSS | 180-day survival rate | In the intention to treat (ITT) analysis, survival insignificantly increased from 71% in the control group to 74% in the CDS group (p = 0.2); in the per protocol (PP) analysis, the survival percentages significantly increased from 71% in the control to 77% in CDS group (p = 0.04). |
| Linares et al, 2011 [48] | 4/10 | Veterans affairs medical centre, USA | Patients who had either a urine culture or a urinalysis that could trigger antibiotic use of UTI | Single-site, uncontrolled before and after | DS within CPOE/EHR | Mean number of days for patients treated for a symptomatic bacteriuria and pyuria | 65% reduction in mean number of antibiotic days (6.3 vs. 2.2 days, p<0.001). |
| Liu et al, 2008 [49] | 4/10 | Tertiary care centre, Taiwan | 858 patients | Single-site, cohort study | DS within CPOE/EHR | The percentage of no prophylactic antibiotic, duration of prophylactic antibiotic, and post-operative wound infection rate | In clean procedures, the percentage of no prophylactic antibiotic decreased in the long run or even reached 100% for some procedures; in clean-contaminated procedures, the duration of prophylactic antibiotic after surgery mostly reduced; the post-operative wound infection rate for clean procedures did not change significantly among the three groups (0.63 vs. 0.72 vs 0.71; p = 0.995). |
| Manjaly et al, 2012 [50] | 4/10 | Teaching hospital, UK | Patients receiving once daily IV gentamicin | Single-site, retrospective analysis | CDSS | Accuracy of dose and frequency of prescription of gentamicin, and time frame for measurement of serum levels | Reductions in prescribing errors in obese patients from 43% to 20%, and non-obese patient; errors in frequency calculation decreased from 12.8% to 4%; gentamicin doses (on average) can be given within 2.5 hours of a blood sample being taken. |
| May et al, 2014 [51] | 4/10 | 311-bed children hospital, USA | Patients whose charts were reviewed 3 years before and 9 months after intervention | Single-site, uncontrolled before and after | DS within CPOE/EHR | Appropriateness of selection and discontinuation of antibiotics | No change in the appropriateness of antibiotic selection and discontinuation (100% vs. 100%). |
| McCluggage et al, 2010 [52] | 6/10 | Tertiary teaching medical centre, USA | Patients with vancomycin orders reviewed 2 months before and 2 months after nomogram introduction | Single-site, uncontrolled before and after | DS within CPOE/EHR | Vancomycin regimen appropriateness, number of regimen changes, and number of serum vancomycin concentration | There was an increase in the appropriateness of vancomycin regimens (23.7% vs.35.8%, p=0.003). No difference was found in number of regimen changes or number of concentration measured. |
| McGregor et al, 2006 [53] | 8/10 | Tertiary care centre, USA | 4507 patients | Single-site RCT | Surveillance system | Hospital antimicrobial expenditure, mortality, length of hospitalization, and time spent managing antimicrobial utilization | Savings of $84,194; $37.64 per patient (hospital antimicrobial expenditures in the intervention and control groups were 285,812 and 372,006, respectively; no significance difference in mortality (3.26% vs. 2.95%, p = 0.55) or length of hospitalisation (3.84 vs. 3.99 days, p = 0.38); the antimicrobial management team spent around one hour less every day on the intervention group. |
| Micek et al, 2014 [54] | 5/10 | Teaching hospital, USA | 3,616 patients | Single-site, cohort study | CDSS | Appropriateness of initial antibiotic treatment, hospital mortality, length of hospital stay, and survival in hospital or ICU | 900 (24.2%) patients were alerted and they were significantly more likely to receive sub-optimal antibiotic therapy (7.1% vs 2.9%, p ˂ 0.001); alerted patients had significantly higher hospital mortality (29.9% vs 23.6, p ˂ 0.001) and hospital length of stay (median, 13.1 vs 10.7 day, p ˂ 0.001) compared with non-alerted patients. |
| Mullet et al, 2001 [55] | 4/10 | 26-bed Paediatric ICU, USA | 1758 patients | Single-site, uncontrolled before-and-after | CDSS | Number of antibiotic orders placed, pharmacist intervention, and sub-therapeutic and excessive antibiotic dosage risk days | Significant reduction in the number of orders placed per anti-infective course by 11.5% (p ˂ 0.01); significant reduction of the rate of pharmacist intervention (59% decrease in the rate of intervention for wrong anti-infective doses and 58% decrease in the rate of clinician requests for dosing help, p ˂ 0.01); significant reduction in the rate of anti-infective sub-therapeutic (36% decrease) and excessive patient days (28% decrease), p ˂ 0.001). |
| Mullet et al, 2004 [56] | 5/10 | Tertiary care centre, USA | 506 patients | Single-site, retrospective study | CDSS | The proportion of appropriate and effective empiric antimicrobial | Empiric antimicrobial therapy suggested by CDS system was significantly more effective than empiric antimicrobial therapy suggested by physicians (86% vs. 66%, p ˂ 0.001). |
| Nachtigall et al, 2014 [57] | 5/10 | Tertiary care centre, Germany | 1316 patients | Single-site uncontrolled before-and-after | CDSS | The percentage of days with adherence guidelines, antibiotic-free days, and morality | Adherence to guideline increased from 61% before intervention to 92% in post 1, decreased in post 2 to 76% and remained significantly higher compared with baseline in post 3, with 71% (p=0.178); antibiotic-free days increased over study periods; at all time periods, mortality for patients with high guideline adherence was lower with 8% versus 12.3% (p=0.014) and an adjusted OR of 1.56 (95% CI 1.05 to 2.31). |
| Paul et al, 2006 [58] | 8/10 | 3 university primary and tertiary care centres in Israel, Germany and Italy | Cohort study: 1203 patients; randomised trial: 2326 patients | Multisite Cohort and RCT | CDSS | Appropriateness of antibiotic treatment cost of observed side effects, duration of hospital stay, and overall 30-day mortality | Cohort: appropriate empirical antibiotic treatment was prescribed by TREAT more significantly and frequently than physicians (70% vs. 57%, p = 0.0001); RCT: the rate of appropriate empirical antibiotic treatment was significantly higher in intervention versus control wards [73% vs. 64%, odds ratio (OR) 1.48, CI: 0.95-2.29]. |
| Pestotnik et al,1996 [59] | 5/10 | Teaching hospital, USA | 162,196 patients | Single site descriptive epidemiologic study. | CDSS | Timing and duration of prophylactic surgical antimicrobials, rates of ADE, patterns of antimicrobial resistance, mortality, length of stay, and DDD/100 bed -days | Increased proportion of hospitalised patients who took antibiotic from 31.8% in 1988 to 53.1% in 1994; decrease in DDD by 22.8%; improved timing of preoperative surgical prophylactic antibiotics from 40% of patients in 1988 to 99.1% in 1994; stabilisation of antimicrobial resistance patterns and length of stay; significant decrease in mortality rate from 3.65% in 1988 to 2.65% in 1994 (p ˂0.001). |
| Pogue et al, 2014 [60] | 8/10 | 3 hospitals, 1100-beds, USA | Patients with Gram-negative bacteraemia (189 patients before and 199 patients after) | Multisite, controlled before and after | Surveillance system | Time to appropriate therapy, length of stay, 30-day mortality | Patients in the intervention group had a lower median time to appropriate therapy than controls (p=0.02) but this was also the case in the pre period (p=0.01). Length of stay was lower in the intervention group (7 vs 8, p<0.001) but the surveillance system had no impact on mortality. |
| Potasman et al, 2012 [61] | 4/10 | 400-bed acute referral hospital, Israel | Patients prescribed antibiotics for which approval is needed | Single-site, uncontrolled before and after | Approval system | Total pharmacy expenditure, expenditure of antibiotics, number of antibiotics requests, rejections by ID team, reasons for rejections | Antibiotic expenditure dropped by 17% (No p-value reported). Over 9000 requests were placed in the system in 1 year, 20.5% were rejected, 43% of those for improper indications. |
| Revolinski et al, 2015 [62] | 6/10 | Tertiary medical centre, USA | 333 patients (172 historical group and 161 intervention group) | Single-centre, uncontrolled before and after | DS within CPOE/EHR | Overall guideline compliance for the treatment of CDIs, utilisation rate of the order set | Compliance with guideline was similar before and after implementation of the Best Practice Alert (BPA) despite significant increase in *C. difficile* order set utilization. Significantly more patients received metronidazole for severe infection and significantly fewer patients with severe complicated infections did not receive combination therapy. |
| Roberts et al, 2010 [63] | 5/10 | Teaching hospital for geriatrics, Australia | 1001 patients | Single-site, uncontrolled  before-and-after | CDSS | The rate of dosing conformity, and appropriateness of therapeutic drug monitoring | Improvements were seen in dosing conformity for gentamicin (63-87%, p=0.01), and vancomycin (47-77%, p=0.07); improvements were seen in therapeutic drug monitoring for gentamicin (70-90%, p=0.02) and vancomycin (61-84%, p=0.17); renally cleared medications were held during acute renal impairment on 62% of instances in the post-intervention compared with 38% pre-intervention, (p=0.01). |
| Rodriguez-Maresca et al, 2014 [64] | 6/10 | Third-level hospital, Spain | 218 patients | Single-site, uncontrolled before-and-after | CDSS | The appropriateness of PMRTR and LRM recommendations | The percentage of appropriateness of the empiric antibiotic treatment was significantly higher when PMRTR and LRMs guidelines were adopted rather than other criteria; LRMs were used for antibiotic prescription in 20.2% of the patients and PMRTRs in 78.2%, and active antibiotics against the finally identified bacteria were prescribed in 80.0% of the former group and 82.4% of the latter. |
| Rohrig et al, 2008 [65] | 8/10 | 14-bed surgical ICU, Germany | Patient data were extracted from EMR/CPOE | Single-site, uncontrolled before and after | DS within CPOE/EMR | Adequate antibiotic therapy, ICU mortality and length of stay | There was an increase in the adequacy of antibiotic treatment from 47.8% pre-DS to 72.5% post-DS. There was a reduction in mortality from 40.9% to 26.5% (p=0.06). No reduction in length of stay was shown. |
| Schulz et al, 2013 [66] | 5/10 | Teaching medical centre, USA | Patients for whom best practice alert (BPA) was issued | Single-site, uncontrolled before-and-after | CDSS | The percentages of accepted, accepted with modification and rejected de-escalation recommendations | A total of 1,285 stewardship BPAs were created; of which 244 (18.9%) were created and acted upon within 72 hours as the main aim of these alerts was de-escalation; 169 BPAs (69%) were accepted, 30 (12%) were accepted with modification and 45 (18%) were rejected. |
| Shojania et al, 1998 [67] | 7/10 | Teaching hospital, USA | 1798 patients | Single-site RCT | DS within CPOE/EHR | The frequency of initiation and renewal of vancomycin therapy and the duration of therapy prescribed | Intervention group had 32% fewer orders (11.3 versus 16.7 orders per physicians, (p = 0.04); 28% fewer patients received initiation or renewal vancomycin orders (7.4 versus 10.3 orders per physicians, p = 0.02); 36% lower duration of vancomycin therapy (26.5 versus 41.2 days, p = 0.05). |
| Sintchenko et al, 2004 [68] | 6/10 | Tertiary hospital, Australia | 31 infectious diseases and critical care clinicians | Multisite cross over trial | CDSS | Adoption rate, decision effectiveness, clinical impact and time to make decision | VAP guidelines were used in 24 (39%) of the 62 cases scenarios, microbiology reports in 36 (58%) and CDSSs in 37 (60%); more time was required to take decision using CDS system (245 seconds) than with unguided methods (113 seconds) (p ˂ 0.001); clinical impact scores for CDS plus laboratory reports, laboratory reports and guidelines were 0.58, 0.45 and 0.26, respectively. |
| Sintchenko et al, 2005 [69] | 6/10 | ICU of 800-bed tertiary hospital, Australia | 12 intensivists and advanced trainees | Single-site, uncontrolled before-and-after | CDSS | DDD/1000 patient-days, length of stay, and mortality | Significant decrease in patients’ length of stay from 7.15 in pre-intervention period to 6.22 bed-days in post-intervention period (p = 0.02); significant reduction in DDDs per 1,000 patient-days of antibiotics (beta-lactams, vancomycin, fluoroquinolones, carbapenems and macrolides) from 1,767 DDD to 1,458 DDDs/1000 patient-days (p= 0.04). |
| Staicu et al, 2016 [70] | 6/10 | 528-bed tertiary referral community Teaching hospital, USA | Patients for whom aztreonam was prescribed at any time during their presentation | Single-site, uncontrolled before-and-after | CDSS | Total aztreonam usage (days of therapy [DOT] per 1000 patient-days) and inappropriate aztreonam usage (DOT per 1000 patient-days) | The total DOT with aztreonam significantly decreased from 9.5 per 1,000 patient-days in the pre-intervention group to 4.4 per 1,000 patient days in the post-intervention group (p<0.0001). The number of inappropriate aztreonam DOT decreased from 4.0 per 1,000 patient days to 0.8 per 1,000 patient-days (p<0.0001) |
| Stevenson et al, 2005 [71] | 5/10 | Five rural hospitals, USA | 240 patients | Multisite, uncontrolled before-and-after | CDSS | Agreement with all recommendations made by CDS, compliance, mortality, 30-day readmission rate, and transfer to another hospital | Agreement of all recommendations had a pooled odds ratio of 1.88 (95% CI, 1.01 to 3.56, p = 0.04), and agreement with recommended dose had a pooled odds ratio of 1.97 (95% CI, 1.04 to 3.74, P = 0.04); overall mortality, 30-day readmission rates, and transfers to another facility were not different between the baseline and intervention time period across all hospitals; compliance with complete study protocol by hospitals ranged from 0% to 71%. |
| Strom et al, 2010 [72] | 5/10 | Two teaching hospitals, USA | 1971 patients | Multisite RCT | DS within CPOE/EHR | Prescribing TPM/SMX with warfarin | The percentage of desired responses by the clinicians was 57% (n=111); the comparable percentage in the control group was 13.5% (n=20); clinicians in the intervention group were less likely than clinicians in the control group to re-order the alert triggering drug (adjusted odd ration, 0.12; 95% CI, 0.045-0.33). |
| Tafelski et al, 2010 [73] | 3/10 | 61-bed ICU at 3200 community acute care university hospital, Germany | 186 patients | Single-site, uncontrolled before-and-after | CDSS | The relationship between adherence to antibiotic recommendations and mortality | ICU mortality was significantly increased in low adherence group (LAG) versus high adherence group (HAG) patients (odds ratio [OR] 2.43, 95% CI 1.126, 5.243); adherence to standards increased significantly by 35% (from 52.3% pre- to 87.2% post-intervention, p < 0.05). |
| Thiel et al, 2009 [74] | 7/10 | 1200-bed teaching medical centre, USA | Patients who are diagnosed with severe sepsis selected for review 6 months before and after implementation | Single-site ,uncontrolled before and after | DS within CPOE/EHR | Appropriateness of antibiotic prescribing, hospital mortality and length of stay | Patients in the post intervention group received more appropriate antibiotics 65.5% vs. 53%, had a shorter length of stay (22.4 vs.28.7 days, p=0.02) and had reduced hospital mortality (47.4% vs 67.4%, p=0.02). |
| Thursky et al, 2006 [75] | 6/10 | 24-bed ICU in adult tertiary hospital, Australia | 986 patients | Single-site, uncontrolled before-and-after | CDSS | Number of courses of antibiotic prescribed, DDD/100 occupied ICU bed days, antibiotic susceptibility mismatches, and system uptake | Significant reduction in proportion of patients prescribed carbapenems, third-generation cephalosporins and vancomycin (OR = 0.61, p = 0.04; OR = 0.58, p = 0.001; OR= 0.67, p = 0.05, respectively); reduction of antibiotic use (from 166 to 149 DDDs/100 ICU bed days); fewer susceptibility mismatches for initial antimicrobial treatment (OR= 0.63, p=0.02). |
| Traugott et al, 2011 [76] | 5/10 | 604-bed acute care teaching hospital, USA | Patients who had vancomycin levels ordered (100 pre and 100 post intervention) | Single-site, uncontrolled before and after | DS within CPOE/EHR | Appropriate vancomycin levels ordered | The level of appropriateness of vancomycin levels increased after DS from 58% to 68%, p=0.02). |
| Van Sise et al, 2012 [77] | 6/10 | Teaching hospital , USA | 2,273 patients | Single-site, uncontrolled before and after | DS within CPOE/EHR | Appropriateness of prophylactic antibiotic treatments (type and dose preoperatively) | Significant increase in the number of women who received adequate prophylactic antibiotic from 85.7% before the intervention to 92.6% after the intervention, p ˂ 0.005; the recommended antibiotics received increased from 43.4% before the intervention to 58.31% after the intervention, p ˂ 0.005. |
| Vincent et al, 2009 [78] | 5/10 | Teaching hospital, USA | 97 patients | Single-site retrospective review study | CDSS | Time to pharmacist completion of PTD request, medication turn-around times, dose adjustment for renal dysfunction, and medication errors | 29 minutes was the median time for pharmacist to complete PTD; delays were seen for the intervention group in comparison to the baseline group for median time to first dose of vancomycin and aminoglycoside (185 vs. 138 min, p = 0.45) and for any antibiotic (134 vs. 118 min, p = 0.42), respectively; significant reduction in medication errors were seen in the intervention group (5 vs. 18 errors, p = 0.002). |
| Westphal et al, 2011 [79] | 5/10 | Teaching hospital, France | Patients for whom 471 pneumonia orders were generated | Single-site, uncontrolled before-and-after | DS within CPOE/EHR | Physician adherence to locally adopted guidelines | The percentage of antibiotic orders containing at least one criterion of non-conformity to the guidelines decreased significantly from 51% pre- to 33% post-intervention (p=0.001); the proportion of non-conform orders for the daily dosage of antibiotics decreased significantly from 26.9% pre- to 12.2% post-intervention (p ˂ 0.001). |
| Yong et al, 2010 [80] | 7/10 | Teaching hospital, Australia | 2838 Gram-negative isolates | Single-site, uncontrolled before-and-after | CDSS | Susceptibility and resistance rate. DDD/1000 bed days, number of admissions, and length of stay. | Significant improvement in susceptibility of *Pseudomonas* to imipenem (18.3% / year, p = 0.009), and gentamicin (11.6% / year, p = 0.02); lower rates of susceptibility of *Enterobacteriaceae* to gentamicin and ciprofloxacin |

CDSS: computerised decision support systems; DS: decision support; EPR: electronic patient record; EHR: electronic patient record; SMX-TMP: sulfamethoxazole-trimethoprim, ITS: interrupted time series; CPOE: computerised physician order; DDD: defined daily dose, ED: emergency department, DOT: days of therapy; LRMs, local resistance maps; PMRTRs, preliminary microbiological reports with therapeutic recommendation

**List A: References for included studies**

1. Agwu AL, Lee CK, Jain SK, [Murray KL](https://www.ncbi.nlm.nih.gov/pubmed/?term=Murray%20KL%5BAuthor%5D&cauthor=true&cauthor_uid=18680419), [Topolski J](https://www.ncbi.nlm.nih.gov/pubmed/?term=Topolski%20J%5BAuthor%5D&cauthor=true&cauthor_uid=18680419), [Miller RE](https://www.ncbi.nlm.nih.gov/pubmed/?term=Miller%20RE%5BAuthor%5D&cauthor=true&cauthor_uid=18680419) et al. A World Wide Web-based antimicrobial stewardship program improves efficiency, communication, and user satisfaction and reduces cost in a tertiary care pediatric medical center. *Clinical infectious diseases: an official publication of the Infectious Diseases Society of America* 2008; **47**: 747-53
2. Arboe B, Laub RR, Kronborg G,Knudsen J D. Evaluation of the decision support system for antimicrobial treatment, TREAT, in an acute medical ward of a university hospital. *International journal of infectious diseases : IJID : official publication of the International Society for Infectious Diseases* 2014; **29**: 156-61
3. Bourdeaux CP, Davies KJ, Thomas MJ, Bewley J S, Gould T H. Using 'nudge' principles for order set design: a before and after evaluation of an electronic prescribing template in critical care. *BMJ quality & safety* 2014; **23**: 382-8.
4. Brady PW, Brinkman WB, Simmons JM, [Yau C](https://www.ncbi.nlm.nih.gov/pubmed/?term=Yau%20C%5BAuthor%5D&cauthor=true&cauthor_uid=24347649), [White CM](https://www.ncbi.nlm.nih.gov/pubmed/?term=White%20CM%5BAuthor%5D&cauthor=true&cauthor_uid=24347649), [Kirkendall ES](https://www.ncbi.nlm.nih.gov/pubmed/?term=Kirkendall%20ES%5BAuthor%5D&cauthor=true&cauthor_uid=24347649) et al. Oral antibiotics at discharge for children with acute osteomyelitis: a rapid cycle improvement project. *BMJ quality & safety* 2014; **23**: 499-507.
5. Buising KL, Thursky KA, Black JF, [MacGregor L](https://www.ncbi.nlm.nih.gov/pubmed/?term=MacGregor%20L%5BAuthor%5D&cauthor=true&cauthor_uid=18667084), [Street AC](https://www.ncbi.nlm.nih.gov/pubmed/?term=Street%20AC%5BAuthor%5D&cauthor=true&cauthor_uid=18667084), [Kennedy MP](https://www.ncbi.nlm.nih.gov/pubmed/?term=Kennedy%20MP%5BAuthor%5D&cauthor=true&cauthor_uid=18667084) et al. Improving antibiotic prescribing for adults with community acquired pneumonia: Does a computerised decision support system achieve more than academic detailing alone?--A time series analysis. *BMC medical informatics and decision making* 2008; **8**: 35.
6. Buising KL, Thursky KA, Robertson MB, [Black JF](https://www.ncbi.nlm.nih.gov/pubmed/?term=Black%20JF%5BAuthor%5D&cauthor=true&cauthor_uid=18550680), [Street AC](https://www.ncbi.nlm.nih.gov/pubmed/?term=Street%20AC%5BAuthor%5D&cauthor=true&cauthor_uid=18550680), [Richards MJ](https://www.ncbi.nlm.nih.gov/pubmed/?term=Richards%20MJ%5BAuthor%5D&cauthor=true&cauthor_uid=18550680) et al. Electronic antibiotic stewardship--reduced consumption of broad-spectrum antibiotics using a computerized antimicrobial approval system in a hospital setting. *The Journal of antimicrobial chemotherapy* 2008; **62**: 608-16.
7. Burke JP, Pestotnik SL. Antibiotic use and microbial resistance in intensive care units: impact of computer-assisted decision support. *Journal of chemotherapy (Florence, Italy)* 1999; **11**: 530-5.
8. Burton ME, Ash CL, Hill DP Jr., [Handy T](https://www.ncbi.nlm.nih.gov/pubmed/?term=Handy%20T%5BAuthor%5D&cauthor=true&cauthor_uid=1905602), [Shepherd MD](https://www.ncbi.nlm.nih.gov/pubmed/?term=Shepherd%20MD%5BAuthor%5D&cauthor=true&cauthor_uid=1905602), [Vasko MR](https://www.ncbi.nlm.nih.gov/pubmed/?term=Vasko%20MR%5BAuthor%5D&cauthor=true&cauthor_uid=1905602). A controlled trial of the cost benefit of computerized bayesian aminoglycoside administration. *Clinical pharmacology and therapeutics* 1991; **49**: 685-94.
9. Caplinger C, Smith G, Remington R, Madaras-Kelly K. Evaluation of a Computerized Decision Support Intervention to Decrease Use of Anti-Pseudomonal Carbapenems in Penicillin Allergic Patients. *Antibiotics (Basel, Switzerland)* 2016; **5**.
10. Chan ALF, Wang HY, Leung HWC. Incorporation of a gentamicin dosage calculator into a computerized prescriber-order-entry system. *American Journal of Health-System Pharmacy* 2006; **63**: 1344-5.
11. Chan YY, Lin TY, Huang CT, [Deng ST](https://www.ncbi.nlm.nih.gov/pubmed/?term=Deng%20ST%5BAuthor%5D&cauthor=true&cauthor_uid=21982143), [Wu TL](https://www.ncbi.nlm.nih.gov/pubmed/?term=Wu%20TL%5BAuthor%5D&cauthor=true&cauthor_uid=21982143), [Leu HS](https://www.ncbi.nlm.nih.gov/pubmed/?term=Leu%20HS%5BAuthor%5D&cauthor=true&cauthor_uid=21982143) et al. Implementation and outcomes of a hospital-wide computerised antimicrobial stewardship programme in a large medical centre in Taiwan. *International journal of antimicrobial agents* 2011; **38**: 486-92.
12. Chow AL, Lye DC, Arah OA. Mortality Benefits of Antibiotic Computerised Decision Support System: Modifying Effects of Age. *Scientific reports* 2015; **5**: 17346.
13. Chow AL, Ang A, Chow CZ, [Ng TM](https://www.ncbi.nlm.nih.gov/pubmed/?term=Ng%20TM%5BAuthor%5D&cauthor=true&cauthor_uid=26774157), [Teng C](https://www.ncbi.nlm.nih.gov/pubmed/?term=Teng%20C%5BAuthor%5D&cauthor=true&cauthor_uid=26774157), [Ling LM](https://www.ncbi.nlm.nih.gov/pubmed/?term=Ling%20LM%5BAuthor%5D&cauthor=true&cauthor_uid=26774157) et al. Implementation hurdles of an interactive, integrated, point-of-care computerised decision support system for hospital antibiotic prescription. *International journal of antimicrobial agents* 2016; **47**: 132-9.
14. Cook PP, Rizzo S, Gooch M, [Jordan M](https://www.ncbi.nlm.nih.gov/pubmed/?term=Jordan%20M%5BAuthor%5D&cauthor=true&cauthor_uid=21059617), [Fang X](https://www.ncbi.nlm.nih.gov/pubmed/?term=Fang%20X%5BAuthor%5D&cauthor=true&cauthor_uid=21059617), [Hudson S](https://www.ncbi.nlm.nih.gov/pubmed/?term=Hudson%20S%5BAuthor%5D&cauthor=true&cauthor_uid=21059617). Sustained reduction in antimicrobial use and decrease in methicillin-resistant Staphylococcus aureus and Clostridium difficile infections following implementation of an electronic medical record at a tertiary-care teaching hospital. *Journal of Antimicrobial Chemotherapy* 2011; **66**: 205-9.
15. Cox ZL, Nelsen CL, Waitman LR, McCoy J A, Peterson J F. Effects of clinical decision support on initial dosing and monitoring of tobramycin and amikacin. *Am J Health Syst Pharm* 2011; **68**: 624-32.
16. Dean NC, Jones BE, Jones JP, [Ferraro JP](https://www.ncbi.nlm.nih.gov/pubmed/?term=Ferraro%20JP%5BAuthor%5D&cauthor=true&cauthor_uid=25725592), [Post HB](https://www.ncbi.nlm.nih.gov/pubmed/?term=Post%20HB%5BAuthor%5D&cauthor=true&cauthor_uid=25725592), [Aronsky D](https://www.ncbi.nlm.nih.gov/pubmed/?term=Aronsky%20D%5BAuthor%5D&cauthor=true&cauthor_uid=25725592) et al. Impact of an Electronic Clinical Decision Support Tool for Emergency Department Patients With Pneumonia. *Annals of emergency medicine* 2015; **66**: 511-20.
17. Demonchy E, Dufour JC, Gaudart J, [Cervetti E](https://www.ncbi.nlm.nih.gov/pubmed/?term=Cervetti%20E%5BAuthor%5D&cauthor=true&cauthor_uid=24898019), [Michelet P](https://www.ncbi.nlm.nih.gov/pubmed/?term=Michelet%20P%5BAuthor%5D&cauthor=true&cauthor_uid=24898019), [Poussard N](https://www.ncbi.nlm.nih.gov/pubmed/?term=Poussard%20N%5BAuthor%5D&cauthor=true&cauthor_uid=24898019) et al. Impact of a computerized decision support system on compliance with guidelines on antibiotics prescribed for urinary tract infections in emergency departments: a multicentre prospective before-and-after controlled interventional study. *J Antimicrob Chemother* 2014; **69**: 2857-63.
18. Devabhakthuni S, Gonzales J, Tata A, Lee S, Shah P, Ibe Offurum A et al. Evaluation of Vancomycin Dosing and Monitoring in Adult Medicine Patients. *Hospital Pharmacy* 2012; **47**: 451-9.
19. Diasinos N, Baysari M, Kumar S et al. Does the availability of therapeutic drug monitoring, computerised dose recommendation and prescribing decision support services promote compliance with national gentamicin prescribing guidelines? *Internal medicine journal* 2015; **45**: 55-62.
20. Evans RS, Pestotnik SL, Burke JP, [Gardner RM](https://www.ncbi.nlm.nih.gov/pubmed/?term=Gardner%20RM%5BAuthor%5D&cauthor=true&cauthor_uid=2327113), [Larsen RA](https://www.ncbi.nlm.nih.gov/pubmed/?term=Larsen%20RA%5BAuthor%5D&cauthor=true&cauthor_uid=2327113), [Classen DC](https://www.ncbi.nlm.nih.gov/pubmed/?term=Classen%20DC%5BAuthor%5D&cauthor=true&cauthor_uid=2327113). Reducing the duration of prophylactic antibiotic use through computer monitoring of surgical patients. *DICP : the annals of pharmacotherapy* 1990; **24**: 351-4.
21. Evans RS, Classen DC, Pestotnik SL, Lundesgaarde H P, Burke J P. Improving empiric antibiotic selection using computer decision support. *Archives of internal medicine* 1994; **154**: 878-84.
22. Evans RS, Classen DC, Pestotnik SL, [Clemmer TP](https://www.ncbi.nlm.nih.gov/pubmed/?term=Clemmer%20TP%5BAuthor%5D&cauthor=true&cauthor_uid=8563367), [Weaver LK](https://www.ncbi.nlm.nih.gov/pubmed/?term=Weaver%20LK%5BAuthor%5D&cauthor=true&cauthor_uid=8563367), [Burke JP](https://www.ncbi.nlm.nih.gov/pubmed/?term=Burke%20JP%5BAuthor%5D&cauthor=true&cauthor_uid=8563367). A decision support tool for antibiotic therapy. *Proceedings Symposium on Computer Applications in Medical Care* 1995: 651-5.
23. Evans RS, Pestotnik SL, Classen DC, Clemmer T P, Weaver LK. A computer-assisted management program for antibiotics and other antiinfective agents. *The New England journal of medicine* 1998; **338**: 232-8.
24. Evans RS, Pestotnik SL, Classen DC [Clemmer TP](https://www.ncbi.nlm.nih.gov/pubmed/?term=Clemmer%20TP%5BAuthor%5D&cauthor=true&cauthor_uid=9435330), [Weaver LK](https://www.ncbi.nlm.nih.gov/pubmed/?term=Weaver%20LK%5BAuthor%5D&cauthor=true&cauthor_uid=9435330), [Orme JF Jr](https://www.ncbi.nlm.nih.gov/pubmed/?term=Orme%20JF%20Jr%5BAuthor%5D&cauthor=true&cauthor_uid=9435330) et al. Evaluation of a computer-assisted antibiotic-dose monitor. *The Annals of pharmacotherapy* 1999; **33**: 1026-31.
25. Faine B, Mohr N, Harland KK [Rolfes K](https://www.ncbi.nlm.nih.gov/pubmed/?term=Rolfes%20K%5BAuthor%5D&cauthor=true&cauthor_uid=26265968), [Porter B](https://www.ncbi.nlm.nih.gov/pubmed/?term=Porter%20B%5BAuthor%5D&cauthor=true&cauthor_uid=26265968), [Fuller BM](https://www.ncbi.nlm.nih.gov/pubmed/?term=Fuller%20BM%5BAuthor%5D&cauthor=true&cauthor_uid=26265968). Importance of Decision Support Implementation in Emergency Department Vancomycin Dosing. *The western journal of emergency medicine* 2015; **16**: 557-64.
26. Filice GA, Drekonja DM, Thurn JR [Rector TS](https://www.ncbi.nlm.nih.gov/pubmed/?term=Rector%20TS%5BAuthor%5D&cauthor=true&cauthor_uid=23651885), [Hamann GM](https://www.ncbi.nlm.nih.gov/pubmed/?term=Hamann%20GM%5BAuthor%5D&cauthor=true&cauthor_uid=23651885), [Masoud BT](https://www.ncbi.nlm.nih.gov/pubmed/?term=Masoud%20BT%5BAuthor%5D&cauthor=true&cauthor_uid=23651885) et al. Use of a computer decision support system and antimicrobial therapy appropriateness. *Infection control and hospital epidemiology* 2013; **34**: 558-65.
27. Fischer MA, Solomon DH, Teich JM. Conversion from intravenous to oral medications: assessment of a computerized intervention for hospitalized patients. *Arch Intern Med* 2003; **163**: 2585-9.
28. Garner SS, Cox TH, Hill EG, [Irving MG](https://www.ncbi.nlm.nih.gov/pubmed/?term=Irving%20MG%5BAuthor%5D&cauthor=true&cauthor_uid=25836318), [Bissinger RL](https://www.ncbi.nlm.nih.gov/pubmed/?term=Bissinger%20RL%5BAuthor%5D&cauthor=true&cauthor_uid=25836318), [Annibale DJ](https://www.ncbi.nlm.nih.gov/pubmed/?term=Annibale%20DJ%5BAuthor%5D&cauthor=true&cauthor_uid=25836318) et al. Prospective, controlled study of an intervention to reduce errors in neonatal antibiotic orders. *Journal of perinatology : official journal of the California Perinatal Association* 2015; **35**: 631-5.
29. Giuliano KK, Lecardo M, Staul L. Impact of protocol watch on compliance with the surviving sepsis campaign. *American journal of critical care : an official publication, American Association of Critical-Care Nurses* 2011; **20**: 313-21.
30. Grayson ML, Melvani S, Kirsa SW, [Cheung S](https://www.ncbi.nlm.nih.gov/pubmed/?term=Cheung%20S%5BAuthor%5D&cauthor=true&cauthor_uid=15115423), [Korman AM](https://www.ncbi.nlm.nih.gov/pubmed/?term=Korman%20AM%5BAuthor%5D&cauthor=true&cauthor_uid=15115423), [Garrett MK](https://www.ncbi.nlm.nih.gov/pubmed/?term=Garrett%20MK%5BAuthor%5D&cauthor=true&cauthor_uid=15115423) et al. Impact of an electronic antibiotic advice and approval system on antibiotic prescribing in an Australian teaching hospital. *The Medical journal of Australia* 2004; **180**: 455-8.
31. Hall AB, Montero J, Cobian J, Regan T. The effects of an electronic order set on vancomycin dosing in the ED. *The American journal of emergency medicine* 2015; **33**: 92-4.
32. Hamad A, Cavell G, Hinton J,Wade P, Whittlesea C.A pre-postintervention study to evaluate the impact of dose calculators on the accuracy of gentamicin and vancomycin initial doses. *BMJ open* 2015; **5**: e006610.
33. Haynes K, Linkin DR, Fishman NO, [Bilker WB](https://www.ncbi.nlm.nih.gov/pubmed/?term=Bilker%20WB%5BAuthor%5D&cauthor=true&cauthor_uid=21262922), [Strom BL](https://www.ncbi.nlm.nih.gov/pubmed/?term=Strom%20BL%5BAuthor%5D&cauthor=true&cauthor_uid=21262922), [Pifer EA](https://www.ncbi.nlm.nih.gov/pubmed/?term=Pifer%20EA%5BAuthor%5D&cauthor=true&cauthor_uid=21262922) et al. Effectiveness of an information technology intervention to improve prophylactic antibacterial use in the postoperative period. *Journal of the American Medical Informatics Association* 2011; **18**: 164-8.
34. Heininger A, Niemetz AH, Keim M, [Fretschner R](https://www.ncbi.nlm.nih.gov/pubmed/?term=Fretschner%20R%5BAuthor%5D&cauthor=true&cauthor_uid=10395153), [Döring G](https://www.ncbi.nlm.nih.gov/pubmed/?term=D%C3%B6ring%20G%5BAuthor%5D&cauthor=true&cauthor_uid=10395153), [Unertl K](https://www.ncbi.nlm.nih.gov/pubmed/?term=Unertl%20K%5BAuthor%5D&cauthor=true&cauthor_uid=10395153). Implementation of an interactive computer-assisted infection monitoring program at the bedside. *Infection control and hospital epidemiology* 1999; **20**: 444-7.
35. Helmons PJ, Grouls RJ, Roos AN, [Bindels AJ](https://www.ncbi.nlm.nih.gov/pubmed/?term=Bindels%20AJ%5BAuthor%5D&cauthor=true&cauthor_uid=20172878), [Wessels-Basten SJ](https://www.ncbi.nlm.nih.gov/pubmed/?term=Wessels-Basten%20SJ%5BAuthor%5D&cauthor=true&cauthor_uid=20172878), [Ackerman EW](https://www.ncbi.nlm.nih.gov/pubmed/?term=Ackerman%20EW%5BAuthor%5D&cauthor=true&cauthor_uid=20172878) et al. Using a clinical decision support system to determine the quality of antimicrobial dosing in intensive care patients with renal insufficiency. *Quality & safety in health care* 2010; **19**: 22-6.
36. Hermsen ED, VanSchooneveld TC, Sayles H,Rupp M E. Implementation of a clinical decision support system for antimicrobial stewardship. *Infection control and hospital epidemiology* 2012; **33**: 412-5.
37. Hulgan T, Rosenbloom ST, Hargrove F, [Talbert DA](https://www.ncbi.nlm.nih.gov/pubmed/?term=Talbert%20DA%5BAuthor%5D&cauthor=true&cauthor_uid=15367178), [Arbogast PG](https://www.ncbi.nlm.nih.gov/pubmed/?term=Arbogast%20PG%5BAuthor%5D&cauthor=true&cauthor_uid=15367178), [Bansal P](https://www.ncbi.nlm.nih.gov/pubmed/?term=Bansal%20P%5BAuthor%5D&cauthor=true&cauthor_uid=15367178) et al. Oral quinolones in hospitalized patients: an evaluation of a computerized decision support intervention. *Journal of internal medicine* 2004; **256**: 349-57.
38. Hum RS, Cato K, Sheehan B, [Patel S](https://www.ncbi.nlm.nih.gov/pubmed/?term=Patel%20S%5BAuthor%5D&cauthor=true&cauthor_uid=25024755), [Duchon J](https://www.ncbi.nlm.nih.gov/pubmed/?term=Duchon%20J%5BAuthor%5D&cauthor=true&cauthor_uid=25024755), [DeLaMora P](https://www.ncbi.nlm.nih.gov/pubmed/?term=DeLaMora%20P%5BAuthor%5D&cauthor=true&cauthor_uid=25024755) et al. Developing clinical decision support within a commercial electronic health record system to improve antimicrobial prescribing in the neonatal ICU. *Applied clinical informatics* 2014; **5**: 368-87.
39. Hwang HG, Chang IC, Hung WF, Sung M L,Yen D.The design and evaluation of clinical decision support systems in the area of pharmacokinetics. *Medical informatics and the Internet in medicine* 2004; **29**: 239-51.
40. Karsies TJ, Sargel CL, Marquardt DJ, [Tofighi S](https://www.ncbi.nlm.nih.gov/pubmed/?term=Tofighi%20S%5BAuthor%5D&cauthor=true&cauthor_uid=20703588), [Salehi A](https://www.ncbi.nlm.nih.gov/pubmed/?term=Salehi%20A%5BAuthor%5D&cauthor=true&cauthor_uid=20703588), [Amanati A](https://www.ncbi.nlm.nih.gov/pubmed/?term=Amanati%20A%5BAuthor%5D&cauthor=true&cauthor_uid=20703588) et al. An empiric antibiotic protocol using risk stratification improves antibiotic selection and timing in critically ill children. *Annals of the American Thoracic Society* 2014; **11**: 1569-75.
41. Kazemi A, Ellenius J, Pourasghar F, [Tofighi S](https://www.ncbi.nlm.nih.gov/pubmed/?term=Tofighi%20S%5BAuthor%5D&cauthor=true&cauthor_uid=20703588), [Salehi A](https://www.ncbi.nlm.nih.gov/pubmed/?term=Salehi%20A%5BAuthor%5D&cauthor=true&cauthor_uid=20703588), [Amanati A](https://www.ncbi.nlm.nih.gov/pubmed/?term=Amanati%20A%5BAuthor%5D&cauthor=true&cauthor_uid=20703588) et al. The effect of Computerized Physician Order Entry and decision support system on medication errors in the neonatal ward: experiences from an Iranian teaching hospital. *Journal of medical systems* 2011; **35**: 25-37.
42. Kim J, Joo EJ, Ha YE [Park SY](https://www.ncbi.nlm.nih.gov/pubmed/?term=Park%20SY%5BAuthor%5D&cauthor=true&cauthor_uid=23361401), [Kang CI](https://www.ncbi.nlm.nih.gov/pubmed/?term=Kang%20CI%5BAuthor%5D&cauthor=true&cauthor_uid=23361401), [Chung DR](https://www.ncbi.nlm.nih.gov/pubmed/?term=Chung%20DR%5BAuthor%5D&cauthor=true&cauthor_uid=23361401) et al. Impact of a computerized alert system for bacteremia notification on the appropriate antibiotic treatment of Staphylococcus aureus bloodstream infections. *European journal of clinical microbiology & infectious diseases : official publication of the European Society of Clinical Microbiology* 2013; **32**: 937-45.
43. King WJ, Le Saux N, Sampson M, [Gaboury I](https://www.ncbi.nlm.nih.gov/pubmed/?term=Gaboury%20I%5BAuthor%5D&cauthor=true&cauthor_uid=17250764), [Norris M](https://www.ncbi.nlm.nih.gov/pubmed/?term=Norris%20M%5BAuthor%5D&cauthor=true&cauthor_uid=17250764), [Moher D](https://www.ncbi.nlm.nih.gov/pubmed/?term=Moher%20D%5BAuthor%5D&cauthor=true&cauthor_uid=17250764). Effect of point of care information on inpatient management of bronchiolitis. *BMC Pediatrics* 2007; **7**.
44. Kofoed K, Zalounina A, Andersen O, [Lisby G](https://www.ncbi.nlm.nih.gov/pubmed/?term=Lisby%20G%5BAuthor%5D&cauthor=true&cauthor_uid=19091808), [Paul M](https://www.ncbi.nlm.nih.gov/pubmed/?term=Paul%20M%5BAuthor%5D&cauthor=true&cauthor_uid=19091808), [Leibovici L](https://www.ncbi.nlm.nih.gov/pubmed/?term=Leibovici%20L%5BAuthor%5D&cauthor=true&cauthor_uid=19091808) et al. Performance of the TREAT decision support system in an environment with a low prevalence of resistant pathogens. *Journal of Antimicrobial Chemotherapy* 2009; **63**: 400-4.
45. Kweekel DM, le Cessie S, Guchelaar HJ, [Vermeij P](https://www.ncbi.nlm.nih.gov/pubmed/?term=Vermeij%20P%5BAuthor%5D&cauthor=true&cauthor_uid=15540484), [Van den Broek PJ](https://www.ncbi.nlm.nih.gov/pubmed/?term=Van%20den%20Broek%20PJ%5BAuthor%5D&cauthor=true&cauthor_uid=15540484), [Twiss IM](https://www.ncbi.nlm.nih.gov/pubmed/?term=Twiss%20IM%5BAuthor%5D&cauthor=true&cauthor_uid=15540484). et al. Outcomes of an antimicrobial control program in a Dutch hospital. *American journal of health-system pharmacy : AJHP : official journal of the American Society of Health-System Pharmacists* 2004; **61**: 1702-6.
46. Larsen RA, Evans RS, Burke JP [Pestotnik SL](https://www.ncbi.nlm.nih.gov/pubmed/?term=Pestotnik%20SL%5BAuthor%5D&cauthor=true&cauthor_uid=2745959), [Gardner RM](https://www.ncbi.nlm.nih.gov/pubmed/?term=Gardner%20RM%5BAuthor%5D&cauthor=true&cauthor_uid=2745959), [Classen DC](https://www.ncbi.nlm.nih.gov/pubmed/?term=Classen%20DC%5BAuthor%5D&cauthor=true&cauthor_uid=2745959). Improved Perioperative Antibiotic Use and Reduced Surgical Wound Infections Through Use of Computer Decision Analysis. *Infection Control & Hospital Epidemiology* 1989; **10**: 316-20.
47. Leibovici L, Gitelman V, Yehezkelli Y, [Poznanski O](https://www.ncbi.nlm.nih.gov/pubmed/?term=Poznanski%20O%5BAuthor%5D&cauthor=true&cauthor_uid=9408069), [Milo G](https://www.ncbi.nlm.nih.gov/pubmed/?term=Milo%20G%5BAuthor%5D&cauthor=true&cauthor_uid=9408069), [Paul M](https://www.ncbi.nlm.nih.gov/pubmed/?term=Paul%20M%5BAuthor%5D&cauthor=true&cauthor_uid=9408069) et al. Improving empirical antibiotic treatment: prospective, nonintervention testing of a decision support system. *Journal of internal medicine* 1997; **242**: 395-400.
48. Leibovici L, Kariv G, Paul M. Long-term survival in patients included in a randomized controlled trial of treat, a decision support system for antibiotic treatment. *Journal of Antimicrobial Chemotherapy* 2013; **68**: 2664-6.
49. Linares LA, Thornton DJ, Strymish J, Baker E, Gupta K. Electronic memorandum decreases unnecessary antimicrobial use for asymptomatic bacteriuria and culture-negative pyuria. *Infection control and hospital epidemiology* 2011; **32**: 644-8.
50. Liu SA, Chiu YT, Lin WD, Chen S J. Using information technology to reduce the inappropriate use of surgical prophylactic antibiotic. *European archives of oto-rhino-laryngology : official journal of the European Federation of Oto-Rhino-Laryngological Societies (EUFOS) : affiliated with the German Society for Oto-Rhino-Laryngology - Head and Neck Surgery* 2008; **265**: 1109-12.
51. Manjaly JG, Reece-Smith AM, Sivaloganathan SS, [Thuraisamy C](https://www.ncbi.nlm.nih.gov/pubmed/?term=Thuraisamy%20C%5BAuthor%5D&cauthor=true&cauthor_uid=22715426), [Smallwood KL](https://www.ncbi.nlm.nih.gov/pubmed/?term=Smallwood%20KL%5BAuthor%5D&cauthor=true&cauthor_uid=22715426), [Jonas E](https://www.ncbi.nlm.nih.gov/pubmed/?term=Jonas%20E%5BAuthor%5D&cauthor=true&cauthor_uid=22715426) et al. Improving dosing of gentamicin in the obese patient: a 3-cycle drug chart and case note audit. *JRSM short reports* 2012; **3**: 25.
52. May LJ, Longhurst CA, Pageler NM, [Wood MS](https://www.ncbi.nlm.nih.gov/pubmed/?term=Wood%20MS%5BAuthor%5D&cauthor=true&cauthor_uid=24732291), [Sharek PJ](https://www.ncbi.nlm.nih.gov/pubmed/?term=Sharek%20PJ%5BAuthor%5D&cauthor=true&cauthor_uid=24732291), [Zebrack CM](https://www.ncbi.nlm.nih.gov/pubmed/?term=Zebrack%20CM%5BAuthor%5D&cauthor=true&cauthor_uid=24732291). Optimizing care of adults with congenital heart disease in a pediatric cardiovascular ICU using electronic clinical decision support*. *Pediatric critical care medicine : a journal of the Society of Critical Care Medicine and the World Federation of Pediatric Intensive and Critical Care Societies* 2014; **15**: 428-34.
53. McCluggage L, Lee K, Potter T, Dugger R, Pakyz A.Implementation and evaluation of vancomycin nomogram guidelines in a computerized prescriber-order-entry system. *American journal of health-system pharmacy : AJHP : official journal of the American Society of Health-System Pharmacists* 2010; **67**: 70-5.
54. McGregor JC, Weekes E, Forrest GN, [Standiford HC](https://www.ncbi.nlm.nih.gov/pubmed/?term=Standiford%20HC%5BAuthor%5D&cauthor=true&cauthor_uid=16622162), [Perencevich EN](https://www.ncbi.nlm.nih.gov/pubmed/?term=Perencevich%20EN%5BAuthor%5D&cauthor=true&cauthor_uid=16622162), [Furuno JP](https://www.ncbi.nlm.nih.gov/pubmed/?term=Furuno%20JP%5BAuthor%5D&cauthor=true&cauthor_uid=16622162) et al. Impact of a computerized clinical decision support system on reducing inappropriate antimicrobial use: a randomized controlled trial. *J Am Med Inform Assoc* 2006; **13**: 378-84.
55. Micek ST, Heard KM, Gowan M, Kollef M H. Identifying critically ill patients at risk for inappropriate antibiotic therapy: a pilot study of a point-of-care decision support alert. *Critical care medicine* 2014; **42**: 1832-8.
56. Mullett CJ, Evans RS, Christenson JC, Dean M J. Development and impact of a computerized pediatric antiinfective decision support program. *Pediatrics* 2001; **108**: E75.
57. Mullett CJ, Thomas JG, Smith CL,Sarwari A R, Khakoo R A. Computerized antimicrobial decision support: an offline evaluation of a database-driven empiric antimicrobial guidance program in hospitalized patients with a bloodstream infection. *Int J Med Inform* 2004; **73**: 455-60.
58. Nachtigall I, Tafelski S, Deja M, [Halle E](https://www.ncbi.nlm.nih.gov/pubmed/?term=Halle%20E%5BAuthor%5D&cauthor=true&cauthor_uid=25534209), [Grebe MC](https://www.ncbi.nlm.nih.gov/pubmed/?term=Grebe%20MC%5BAuthor%5D&cauthor=true&cauthor_uid=25534209), [Tamarkin A](https://www.ncbi.nlm.nih.gov/pubmed/?term=Tamarkin%20A%5BAuthor%5D&cauthor=true&cauthor_uid=25534209) et al. Long-term effect of computer-assisted decision support for antibiotic treatment in critically ill patients: a prospective 'before/after' cohort study. *BMJ Open* 2014; **4**: e005370.
59. Paul M, Andreassen S, Tacconelli E, [Nielsen AD](https://www.ncbi.nlm.nih.gov/pubmed/?term=Nielsen%20AD%5BAuthor%5D&cauthor=true&cauthor_uid=16998208), [Almanasreh N](https://www.ncbi.nlm.nih.gov/pubmed/?term=Almanasreh%20N%5BAuthor%5D&cauthor=true&cauthor_uid=16998208), [Frank U](https://www.ncbi.nlm.nih.gov/pubmed/?term=Frank%20U%5BAuthor%5D&cauthor=true&cauthor_uid=16998208) et al. Improving empirical antibiotic treatment using TREAT, a computerized decision support system: cluster randomized trial. *The Journal of antimicrobial chemotherapy* 2006; **58**: 1238-45.
60. Pestotnik SL, Classen DC, Evans RS, Burke J P. Implementing antibiotic practice guidelines through computer-assisted decision support: clinical and financial outcomes. *Annals of internal medicine* 1996; **124**: 884-90.
61. Pogue JM, Mynatt RP, Marchaim D, [Zhao JJ](https://www.ncbi.nlm.nih.gov/pubmed/?term=Zhao%20JJ%5BAuthor%5D&cauthor=true&cauthor_uid=24442074), [Barr VO](https://www.ncbi.nlm.nih.gov/pubmed/?term=Barr%20VO%5BAuthor%5D&cauthor=true&cauthor_uid=24442074), [Moshos J](https://www.ncbi.nlm.nih.gov/pubmed/?term=Moshos%20J%5BAuthor%5D&cauthor=true&cauthor_uid=24442074) et al. Automated alerts coupled with antimicrobial stewardship intervention lead to decreases in length of stay in patients with gram-negative bacteremia. *Infection control and hospital epidemiology* 2014; **35**: 132-8.
62. Potasman I, Naftali G, Grupper M. Impact of a computerized integrated antibiotic authorization system. *Israel Medical Association Journal* 2012; **14**: 415-9.
63. Revolinski S. Implementation of a Clinical Decision Support Alert for the Management of Clostridium difficile Infection. *Antibiotics (Basel, Switzerland)* 2015; **4**: 667-74.
64. Roberts GW, Farmer CJ, Cheney PC, [Govis SM](https://www.ncbi.nlm.nih.gov/pubmed/?term=Govis%20SM%5BAuthor%5D&cauthor=true&cauthor_uid=20442149), [Belcher TW](https://www.ncbi.nlm.nih.gov/pubmed/?term=Belcher%20TW%5BAuthor%5D&cauthor=true&cauthor_uid=20442149), [Walsh SA](https://www.ncbi.nlm.nih.gov/pubmed/?term=Walsh%20SA%5BAuthor%5D&cauthor=true&cauthor_uid=20442149) et al. Clinical decision support implemented with academic detailing improves prescribing of key renally cleared drugs in the hospital setting. *J Am Med Inform Assoc* 2010; **17**: 308-12.
65. Rodriguez-Maresca M, Sorlozano A, Grau M, [Rodriguez-Castaño R](https://www.ncbi.nlm.nih.gov/pubmed/?term=Rodriguez-Casta%C3%B1o%20R%5BAuthor%5D&cauthor=true&cauthor_uid=25197643), [Ruiz-Valverde A](https://www.ncbi.nlm.nih.gov/pubmed/?term=Ruiz-Valverde%20A%5BAuthor%5D&cauthor=true&cauthor_uid=25197643), [Gutierrez-Fernandez J](https://www.ncbi.nlm.nih.gov/pubmed/?term=Gutierrez-Fernandez%20J%5BAuthor%5D&cauthor=true&cauthor_uid=25197643). Implementation of a computerized decision support system to improve the appropriateness of antibiotic therapy using local microbiologic data. *BioMed research international* 2014; **2014**: 395434.
66. Rohrig R, Niczko EJ, Beutefuhr H, [Böttger S](https://www.ncbi.nlm.nih.gov/pubmed/?term=B%C3%B6ttger%20S%5BAuthor%5D&cauthor=true&cauthor_uid=18487709), [Klasen J](https://www.ncbi.nlm.nih.gov/pubmed/?term=Klasen%20J%5BAuthor%5D&cauthor=true&cauthor_uid=18487709), [Füssle R](https://www.ncbi.nlm.nih.gov/pubmed/?term=F%C3%BCssle%20R%5BAuthor%5D&cauthor=true&cauthor_uid=18487709) et al. Examination of computer assisted prescribing of an initial calculated antibiotic treatment. *Studies in health technology and informatics* 2008; **136**: 63-8.
67. Schulz L, Osterby K, Fox B. The use of best practice alerts with the development of an antimicrobial stewardship navigator to promote antibiotic de-escalation in the electronic medical record. *Infection control and hospital epidemiology* 2013; **34**: 1259-65.
68. Shojania KG, Yokoe D, Platt R, [Fiskio J](https://www.ncbi.nlm.nih.gov/pubmed/?term=Fiskio%20J%5BAuthor%5D&cauthor=true&cauthor_uid=9824802), [Ma'luf N](https://www.ncbi.nlm.nih.gov/pubmed/?term=Ma'luf%20N%5BAuthor%5D&cauthor=true&cauthor_uid=9824802), [Bates DW](https://www.ncbi.nlm.nih.gov/pubmed/?term=Bates%20DW%5BAuthor%5D&cauthor=true&cauthor_uid=9824802). Reducing vancomycin use utilizing a computer guideline: results of a randomized controlled trial. *J Am Med Inform Assoc* 1998; **5**: 554-62.
69. Sintchenko V, Coiera E, Iredell JR, Gilbert G L. Comparative impact of guidelines, clinical data, and decision support on prescribing decisions: an interactive web experiment with simulated cases. *Journal of the American Medical Informatics Association*; **11**: 71-7.
70. Sintchenko V, Iredell JR, Gilbert GL, Coiera E. Handheld computer-based decision support reduces patient length of stay and antibiotic prescribing in critical care. *Journal of the American Medical Informatics Association : JAMIA* 2005; **12**: 398-402.
71. Staicu ML, Brundige ML, Ramsey A, [Brown J](https://www.ncbi.nlm.nih.gov/pubmed/?term=Brown%20J%5BAuthor%5D&cauthor=true&cauthor_uid=26896502), [Yamshchikov A](https://www.ncbi.nlm.nih.gov/pubmed/?term=Yamshchikov%20A%5BAuthor%5D&cauthor=true&cauthor_uid=26896502), [Peterson DR](https://www.ncbi.nlm.nih.gov/pubmed/?term=Peterson%20DR%5BAuthor%5D&cauthor=true&cauthor_uid=26896502) et al. Implementation of a penicillin allergy screening tool to optimize aztreonam use. *American journal of health-system pharmacy : AJHP : official journal of the American Society of Health-System Pharmacists* 2016; **73**: 298-306.
72. Stevenson KB, Barbera J, Moore JW, Samore M H, Houck P. Understanding keys to successful implementation of electronic decision support in rural hospitals: analysis of a pilot study for antimicrobial prescribing. *American journal of medical quality : the official journal of the American College of Medical Quality* 2005; **20**: 313-8.
73. Strom BL, Schinnar R, Aberra F, [Bilker W](https://www.ncbi.nlm.nih.gov/pubmed/?term=Bilker%20W%5BAuthor%5D&cauthor=true&cauthor_uid=20876410), [Hennessy S](https://www.ncbi.nlm.nih.gov/pubmed/?term=Hennessy%20S%5BAuthor%5D&cauthor=true&cauthor_uid=20876410), [Leonard CE](https://www.ncbi.nlm.nih.gov/pubmed/?term=Leonard%20CE%5BAuthor%5D&cauthor=true&cauthor_uid=20876410) et al. Unintended effects of a computerized physician order entry nearly hard-stop alert to prevent a drug interaction: a randomized controlled trial. *Arch Intern Med* 2010; **170**: 1578-83.
74. Tafelski S, Nachtigall I, Deja M, [Tamarkin A](https://www.ncbi.nlm.nih.gov/pubmed/?term=Tamarkin%20A%5BAuthor%5D&cauthor=true&cauthor_uid=21309474), [Trefzer T](https://www.ncbi.nlm.nih.gov/pubmed/?term=Trefzer%20T%5BAuthor%5D&cauthor=true&cauthor_uid=21309474), [Halle E](https://www.ncbi.nlm.nih.gov/pubmed/?term=Halle%20E%5BAuthor%5D&cauthor=true&cauthor_uid=21309474) et al. Computer-assisted decision support for changing practice in severe sepsis and septic shock. *J Int Med Res* 2010; **38**: 1605-16.
75. Thiel SW, Asghar MF, Micek ST, [Reichley RM](https://www.ncbi.nlm.nih.gov/pubmed/?term=Reichley%20RM%5BAuthor%5D&cauthor=true&cauthor_uid=19237883), [Doherty JA](https://www.ncbi.nlm.nih.gov/pubmed/?term=Doherty%20JA%5BAuthor%5D&cauthor=true&cauthor_uid=19237883), [Kollef MH](https://www.ncbi.nlm.nih.gov/pubmed/?term=Kollef%20MH%5BAuthor%5D&cauthor=true&cauthor_uid=19237883). Hospital-wide impact of a standardized order set for the management of bacteremic severe sepsis. *Critical care medicine* 2009; **37**: 819-24.
76. Thursky KA, Buising KL, Bak N, [Macgregor L](https://www.ncbi.nlm.nih.gov/pubmed/?term=Macgregor%20L%5BAuthor%5D&cauthor=true&cauthor_uid=16415039), [Street AC](https://www.ncbi.nlm.nih.gov/pubmed/?term=Street%20AC%5BAuthor%5D&cauthor=true&cauthor_uid=16415039), [Macintyre CR](https://www.ncbi.nlm.nih.gov/pubmed/?term=Macintyre%20CR%5BAuthor%5D&cauthor=true&cauthor_uid=16415039) et al. Reduction of broad-spectrum antibiotic use with computerized decision support in an intensive care unit. *International journal for quality in health care : journal of the International Society for Quality in Health Care / ISQua* 2006; **18**: 224-31.
77. Traugott KA, Maxwell PR, Green K, Frei C, Lewis J S. Effects of therapeutic drug monitoring criteria in a computerized prescriber-order-entry system on the appropriateness of vancomycin level orders. *American journal of health-system pharmacy : AJHP : official journal of the American Society of Health-System Pharmacists* 2011; **68**: 347-52.
78. Van Sise MA, Chappelle J, Figueroa R. Improving the selection of recommended prophylactic antibiotics using an electronic medical record. *Obstetrics and Gynecology* 2012; **120**: 1382-5.
79. Vincent WR, Martin CA, Winstead PS, [Smith KM](https://www.ncbi.nlm.nih.gov/pubmed/?term=Smith%20KM%5BAuthor%5D&cauthor=true&cauthor_uid=18952947), [Gatz J](https://www.ncbi.nlm.nih.gov/pubmed/?term=Gatz%20J%5BAuthor%5D&cauthor=true&cauthor_uid=18952947), [Lewis DA](https://www.ncbi.nlm.nih.gov/pubmed/?term=Lewis%20DA%5BAuthor%5D&cauthor=true&cauthor_uid=18952947). Effects of a pharmacist-to-dose computerized request on promptness of antimicrobial therapy. *Journal of the American Medical Informatics Association : JAMIA* 2009; **16**: 47-53.
80. Westphal JF, Jehl F, Javelot H, Nonnemacher C. Enhanced physician adherence to antibiotic use guidelines through increased availability of guidelines at the time of drug ordering in hospital setting. *Pharmacoepidemiology and drug safety* 2011; **20**: 162-8.
81. Yong MK, Buising KL, Cheng AC, Thursky K A. Improved susceptibility of Gram-negative bacteria in an intensive care unit following implementation of a computerized antibiotic decision support system. *J Antimicrob Chemother* 2010; **65**: 1062-9.
