# Supplementary material for: The effectiveness of computerised decision support on antibiotic use in hospitals: A systematic review
Source: PLoS One. 2017 Aug 24;12(8):e0183062. doi: 10.1371/journal.pone.0183062 (PMC5570266; doi:10.1371/journal.pone.0183062)
Supplement: S1 Appendix — (DOCX) [file pone.0183062.s003.docx]

**S1 Appendix**

**PRISMA Search strategy details**

Database: Ovid MEDLINE(R) In-Process & Other Non-Indexed Citations and Ovid MEDLINE(R) <1946 to Present>

Search Strategy:

--------------------------------------------------------------------------------

1 computer* decision support.mp. (528)

2 computer assisted management system.mp. (2)

3 clinical information system.mp (671)

4 computer decision analysis.mp. (1)

5 diagnostic decision support.mp. (121)

6 diagnostic decision support system.mp. (39)

7 computer* provider order entry.mp. (345)

8 computer* physician order entry.mp. (694)

9 computer* antimicrobial approval system.mp. (3)

10 medical order entry.mp. (1596)

11 electronic health record.mp. (2801)

12 electronic medical record.mp. (3571)

13 electronic patient record.mp. (780)

14 electronic pharmac* record.mp. (4)

15 (electronic alert or automated alert).mp. (78)

16 (electronic reminder or automated reminder).mp. (65)

17 (electronic feedback or automated feedback).mp. (191)

18 web-based decision support.mp. (66)

19 bedside decision support.mp. (11)

20 pharmacy decision support.mp. (5)

21 internet-based decision support.mp. (18)

22 personal digital assistant.mp. (555)

23 computer* expert system.mp. (63)

24 information system technology.mp. (47)

25 electronic health.mp. (11091)

26 order communication system.mp. (11)

27 (EHR or EMR or EPR or PICS or CDSS or CPOE or OCS or HIS or DSS or PDA) .mp. (221458)

28 antimicrobial.mp. (98236)

29 (hospital or secondary care of tertiary care or inpatient?) .mp. (945439)

30 antibiotic.mp. (151764)

31 electronic prescribing .mp (846)

32 (case report or comment or congresses or editorial or historical articles or interview or letter or news).pt. (1662041)

33 1 or 2 or 3 or 4 or 5 or 6 or 7 or 8 or 9 or 10 or 11 or 12 or 13 or 14 or 15 or 16 or 17 or 18 or 19 or 20 or 21 or 22 or 23 or 24 or 25 or 26 or 27 or 31 (234990)

34 28 or 30 (233321)

35 antibacterial.mp. (45465)

36 (animals not humans).sh. (4004891)

37 32 or 36 (5601767)

38 29 and 33 and 34 (515)

39 38 not 37 (509)
